# Genetic orchestration of the primary central nervous system lymphoma microenvironment

**DOI:** 10.1186/s40364-026-01004-z

**Published:** 2026-09-29

**Authors:** Xiyue Xu, Xinyi Huang, Cong Li, Jialiang Lu, Xi Chen, Haifeng Yu, Zucheng Xie, Tao Lei, Xiaowu Dong, Haiyan Yang

**Affiliations:** 1 Zhejiang Cancer Hospital, Hangzhou, Zhejiang 310022 China; 2 Wenzhou Medical University, Wenzhou, Zhejiang 325035 China; 3 College of Pharmaceutical Sciences, Zhejiang University, Hangzhou, 310058 China

**Keywords:** Primary central nervous system lymphoma, Genetic alterations, Tumor microenvironment, Targeted therapy

## Abstract

Primary central nervous system lymphoma (PCNSL) of the CNS is a distinct entity of diffuse large B-cell lymphoma (DLBCL). CNS lymphomas primarily occur in immune-privileged sites and tend to be confined to extranodal locations such as the brain, spinal cord, meninges, and/or eyes. Due to the presence of the blood-brain barrier and the inherent “immune privilege” characteristics of brain tissue, the tumor microenvironment (TME) of PCNSL exhibits unique features. Additionally, genetic factors originating from the lymphoma cells themselves significantly influence the TME. Substantial evidence has demonstrated that PCNSL exhibits highly characteristic molecular genetic alterations, with a mutational profile distinctly different from that of systemic DLBCL. These unique genetic variations collectively shape the malignant biological behavior of PCNSL, including activation of signal transduction, evasion of immune surveillance, and promotion of malignant progression. Here, we synthesize current evidence to conceptualize the genetic-TME axis as a unifying framework for PCNSL biology, wherein tumor-intrinsic genetic alterations and the specialized CNS microenvironment interact bidirectionally to shape immune evasion, tumor evolution, and therapeutic response. We review the distinctive genetic landscape of PCNSL, the cellular and molecular architecture of its TME, and the reciprocal mechanisms linking these compartments during disease progression. Integrating these dimensions may refine molecular stratification, facilitate biomarker-guided therapeutic selection, and inform more rational treatment strategies for PCNSL.

## Background

Primary central nervous system lymphoma (PCNSL) is a malignant disease restricted to the brain, eyes, spinal cord, or leptomeninges, without evidence of systemic involvement. This rare and aggressive lymphoma accounts for about 4% to 6% of all extranodal lymphomas and approximately 4% of newly diagnosed malignant brain tumors [1]. Compared with systemic diffuse large B-cell lymphoma (DLBCL), PCNSL carries a substantially poorer prognosis, with historical median survival often confined to 1–2 years in the absence of intensive therapy.

The malignant progression of PCNSL is inherently tied to its unique biological properties. The predominant pathological type is DLBCL, specifically the non-germinal center B-cell-like (non-GCB) subtype. Recent studies have highlighted a unique genomic alteration signature in PCNSL, which includes mutations, somatic copy number alterations, and structural variations. Many genetic abnormalities have been associated with the MCD subtype (characterized by concurrent *MYD88* and *CD79B* mutations) and the C5 subtype within the DLBCL classification. Recent studies have proposed distinct molecular classifications of PCNSL, namely CS1-CS4 and C1-C4 [2, 3]. Intrinsic genetic changes in lymphoma cells are considered significant drivers of tumor development, progression, and treatment response. Furthermore, recent findings emphasize the critical role of non-tumor cells in the tumor microenvironment, which is intricately linked to the genetic alterations in the tumor cells themselves. A thorough understanding of the genetic landscapes associated with PCNSL, alongside the elucidation of the interactions between gene alterations and microenvironmental dynamics, is essential. Such insights are poised to inform the development of personalized treatment strategies and ultimately improve patient outcomes.

The CNS has traditionally been viewed as an immune-privileged site [4], where immune responses are meticulously regulated by anatomical barriers like the blood-brain barrier (BBB) and specialized resident immune populations, particularly microglia. PCNSL develops and thrives within this tightly controlled microenvironment. The tumor microenvironment (TME) of PCNSL is characterized as a complex three-dimensional network comprised of immune cells, endothelial cells, extracellular matrix (ECM), and various signaling molecules [5]. Compared to nodal DLBCL, the TME in PCNSL exhibits distinct structural and functional features. While tumor-infiltrating lymphocytes (TILs) and tumor-associated macrophages (TAMs) are present, the total number of immune cells is significantly lower than that observed in systemic DLBCL. Paradoxically, despite this lower density of immune infiltrates, the immunosuppressive effect exerted by the PCNSL TME is significantly stronger and more pervasive than that observed in systemic DLBCL. This phenomenon implies that PCNSL does not rely on passive mechanisms of immune evasion. Instead, the tumor actively co-opts the local CNS resident immune cells, particularly microglia and TAMs, and exploits specialized secreted factors to establish a profoundly immune-suppressive shield, illustrating a highly efficient mechanism of immune neutralization favored by the anatomical niche. This aggressive TME manipulation is fundamentally linked to the lymphoma cell’s intrinsic genetic architecture. Key recurrent somatic alterations, including the co-occurrence of activating *MYD88* and *CD79B* mutations, drive persistent activation of the NF-κB signaling pathway. This leads to the continuous release of specific cytokines and chemokines that not only support lymphoma survival but also induce a pro-tumor, immunosuppressive phenotype in resident immune cells. In addition, recurrent genetic alterations involving 1p13.1, 6p21, 15q21.1, and related loci converge on a coordinated immune-evasion program that compromises antigen presentation and attenuates T-cell-mediated immune surveillance, thereby facilitating tumor persistence within the CNS immune niche.

Rather than representing parallel and largely independent dimensions of PCNSL biology, tumor-intrinsic genetics and the tumor microenvironment are increasingly understood as components of an integrated and dynamic regulatory system. Here, the genetic-TME axis refers to the bidirectional interplay through which recurrent genetic alterations in lymphoma cells remodel the surrounding CNS niche, while microenvironmental signals reciprocally shape tumor-cell fitness, immune adaptation, and therapeutic response. Oncogenic and immune-evasion programs can alter antigen presentation, cytokine and chemokine signaling, myeloid-cell polarization, T-cell surveillance, and metabolic and stromal interactions, thereby establishing a microenvironment that sustains tumor persistence and treatment resistance. In turn, signals derived from microglia, macrophages, lymphocytes, endothelial cells, and other components of the CNS niche can impose selective pressures and reinforce adaptive signaling states within malignant B cells. This framework therefore extends beyond conventional models that consider tumor genetics, immune dysregulation, or therapeutic vulnerability in isolation, instead emphasizing their mechanistic interdependence within the unique CNS ecosystem. From this perspective, PCNSL can be viewed as a disease shaped not only by cell-intrinsic oncogenic lesions but also by their capacity to reprogram, and subsequently exploit, the surrounding microenvironment. Integrating these two dimensions provides a unifying framework for understanding disease evolution, immune escape, and treatment resistance, while also revealing context-dependent vulnerabilities that may support molecularly informed and microenvironment-directed therapeutic strategies.

## Cell of origin and classification

### Immunophenotypic characterization: establishing and refining the activated B-cell phenotype

Gene expression profiling (GEP) classifies DLBCL into germinal center B cell-like (GCB) and activated B cell-like (ABC) subtypes [6]. Application of the Hans algorithm indicates that approximately 90%-95% of PCNSL cases fall within the non-GCB category, in striking contrast to the more balanced distribution of GCB and ABC subtypes observed in systemic DLBCL, underscoring the distinct molecular identity of PCNSL [1, 7]. However, high-resolution transcriptomic platforms, including NanoString-based digital gene expression profiling, have demonstrated that immunohistochemistry (IHC)-based classification overestimates the prevalence of the ABC subtype, with 10%-15% of tumors initially classified as non-GCB subsequently reassigned to the GCB or unclassified categories following molecular profiling [8–10]. Despite this refinement, the vast majority of PCNSLs retain an ABC-like transcriptional program characterized by recurrent *MYD88* and *CD79B* mutations together with constitutive NF-κB activation [11, 12]. Importantly, PCNSL should not be regarded as a canonical ABC lymphoma. Instead, its characteristic co-expression of BCL6 [13], a germinal center marker, with the post-germinal center regulators IRF4/MUM1 defines a hybrid transcriptional state [7, 14], suggesting that its cell of origin corresponds to a late germinal center B cell undergoing transition toward plasmablastic differentiation rather than a conventional peripheral activated B cell.

### Developmental stage refinement: post-germinal center differentiation arrest

**Fig. 1:**
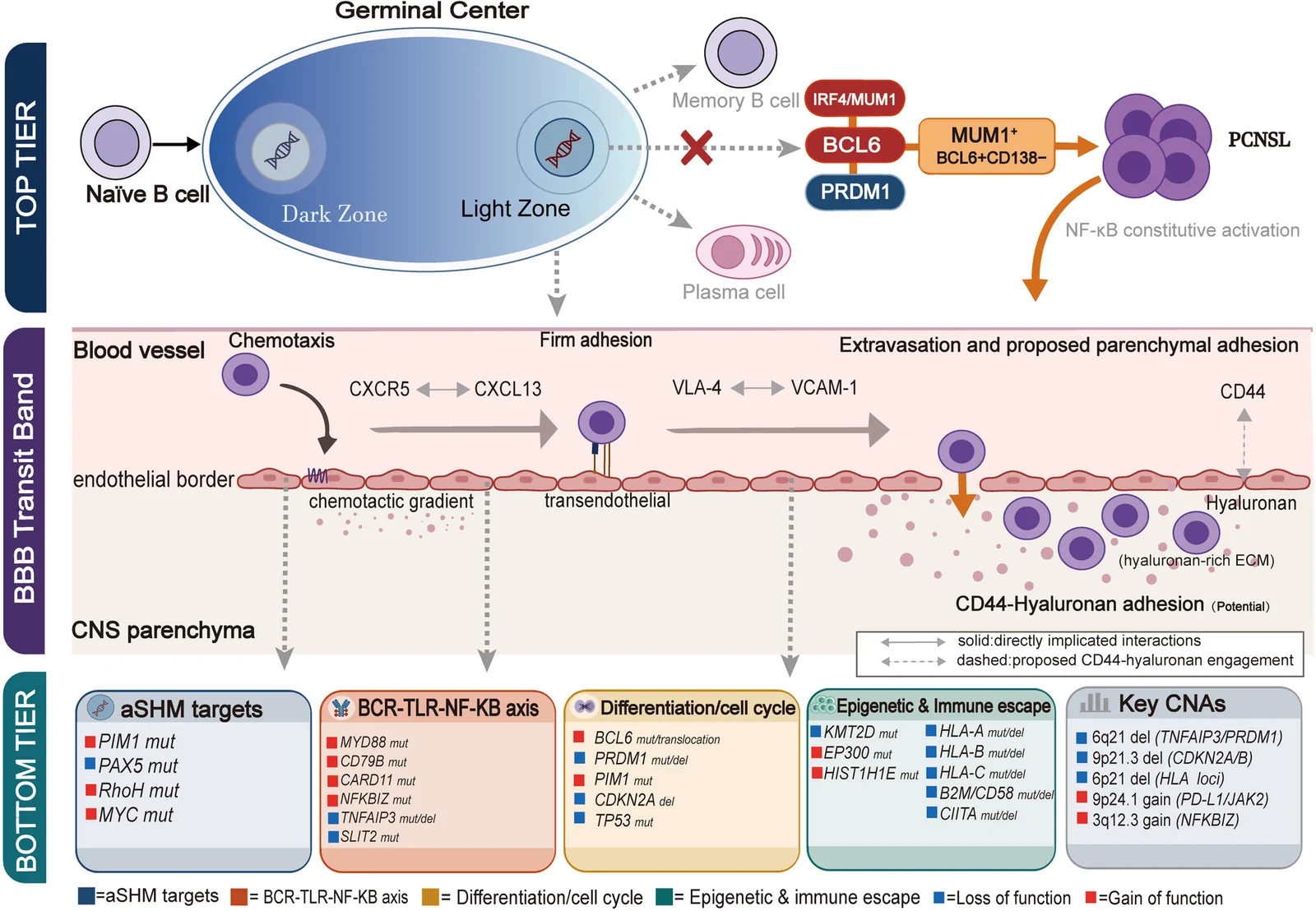
A genotype-driven model of CNS colonization and microenvironmental remodeling in PCNSL. Top Tier: Oncogenic lesions (BCL6 activation, PRDM1 loss) during germinal center transit block terminal differentiation, arresting malignant B cells in a MUM1⁺/BCL6⁺/CD138⁻ state with chronic NF-κB activation. BBB Transit Band: Neoplastic clones cross the blood-brain barrier via CXCL13-CXCR5 chemotaxis, VLA-4-VCAM-1 and CD44-Hyaluronan (potential) adhesion networks. Bottom Tier: Following CNS entry, aberrant somatic hypermutation, genetic variation, and epigenetic remodeling converge to sculpt an immune-evasive and stroma-supported niche that sustains tumor persistence. mut, mutation; del, deletion; CNAs, copy number alterations; aSHM, aberrant somatic hypermutation

Although PCNSL clusters with ABC-DLBCL by gene expression profiling, its cell of origin resides at the late stage of the GC reaction rather than in the peripheral activated B-cell compartment [1]. PCNSL cells exhibit extensive immunoglobulin heavy-chain variable region (IGHV) somatic hypermutation (SHM) together with evidence of ongoing SHM activity, indicating transit through the germinal center (GC) [15]. However, unlike normal post-GC B cells, which extinguish the GC transcriptional program and undergo terminal plasma cell differentiation, PCNSL cells remain locked in a late GC developmental state, failing to complete the differentiation program. BCL6, the master regulator of the GC transcriptional program, is constitutively activated through chromosomal translocations or promoter mutations, thereby sustaining proliferative capacity while preventing GC exit and terminal B-cell differentiation [15, 16]. Concurrently, *PRDM1* (encoding BLIMP1), the key initiator of plasma cell differentiation, is frequently inactivated through loss-of-function mutations or heterozygous deletions, blocking terminal differentiation [2, 15, 17]. This molecular impasse locks tumor cells in a MUM1⁺/BCL6⁺/CD138⁻ transitional state from which chronic BCR/TLR signaling drives malignant expansion (Fig. 1) [14, 15].

### Autoantigen drive and BCR selection: clonal evolution of autoreactive B cells

PCNSL pathogenesis is closely linked to BCR-mediated chronic antigen stimulation, with tumor cells arising from autoreactive B-cell clones that have undergone GC selection and been selectively retained [18]. Immunogenetic analyses reveal a marked *IGHV* usage bias in PCNSL, particularly *IGHV4-34* (frequency 36%) [15], whose encoded antibodies possess the intrinsic structural capacity to recognize self-antigens such as I/i carbohydrate antigens. The *IGHV4-34* mutation may reduce the ability to bind I/i carbohydrate antigens. This specific motif has been implied to be responsible for binding the N-acetylgalactosamine antigenic determinant cluster, suggesting that tumor cells may retain the ability to bind to and be activated by superantigens [15]. Unlike certain hematologic malignancies, such as chronic lymphocytic leukemia (CLL), in which a subset of cases is characterized by stereotyped B-cell receptor immunoglobulins (BcR IGs) [19], PCNSL exhibits broadly multireactive BCR repertoires capable of recognizing CNS-associated autoantigens, including GRINL1A, BAIAP2, and Centaurin-α, as well as the microenvironment-enriched protein Galectin-3 [20]. SAMD14 (Asn339) and Neurabin-I (Asn1277) acquire hyper-N-glycosylation modifications that render them recognizable as neoantigens by tumor BCRs, providing sustained BCR-NF-κB survival input [20, 21].

### Homing mechanism: CNS tropism of the cell of origin

PCNSL has traditionally been viewed as arising from peripheral B-cell clones that acquire the capacity to home to and colonize the CNS, rather than from malignant transformation of resident CNS cells [22, 23]. However, the precise cellular origin of these malignant cells remains unresolved. Two competing models have been proposed: one posits local transformation of peripheral B cells following CNS migration, whereas the other suggests that B cells recruited into the CNS during immune responses, such as infection-associated inflammation, may subsequently undergo neoplastic transformation [1]. Dissecting the relative contribution of these pathways remains an important area for future investigation. Its angiocentric growth pattern supports hematogenous entry across the BBB. This process is orchestrated by the CXCL13-CXCR5 chemotactic axis and complementary adhesion networks [23]. Ectopic CXCL13 expression by CNS endothelial and tumor cells establishes a chemotactic gradient that recruits CXCR5-expressing malignant B cells [24], while activated VLA-4 (α4β1 integrin) engages endothelial VCAM-1 to mediate firm adhesion and subsequent transendothelial migration. Following BBB traversal, the CD44-hyaluronan axis may further facilitate parenchymal dissemination (Fig. 1). CD44 is highly expressed on perivascular PCNSL cells, with overall expression exceeding that observed in systemic DLBCL [25, 26]. By engaging its ligand hyaluronan, CD44 can mediate lymphoma-cell adhesion to CNS white matter, a feature consistent with the preferential white-matter involvement and diffuse infiltrative growth pattern of brain lymphoma [27, 28]. Together, these observations implicate CD44-hyaluronan interactions as a potential contributor to PCNSL parenchymal dissemination, although this mechanism remains to be directly validated in an intact in vivo PCNSL model. Collectively, they support a model in which the PCNSL cell of origin represents a differentiation-arrested B-cell clone that has acquired enhanced CNS-homing and tissue-invasive capacity.

## Characterization and composition of the immune microenvironment of PCNSL

### Spatial organization and cellular composition

**Fig. 2:**
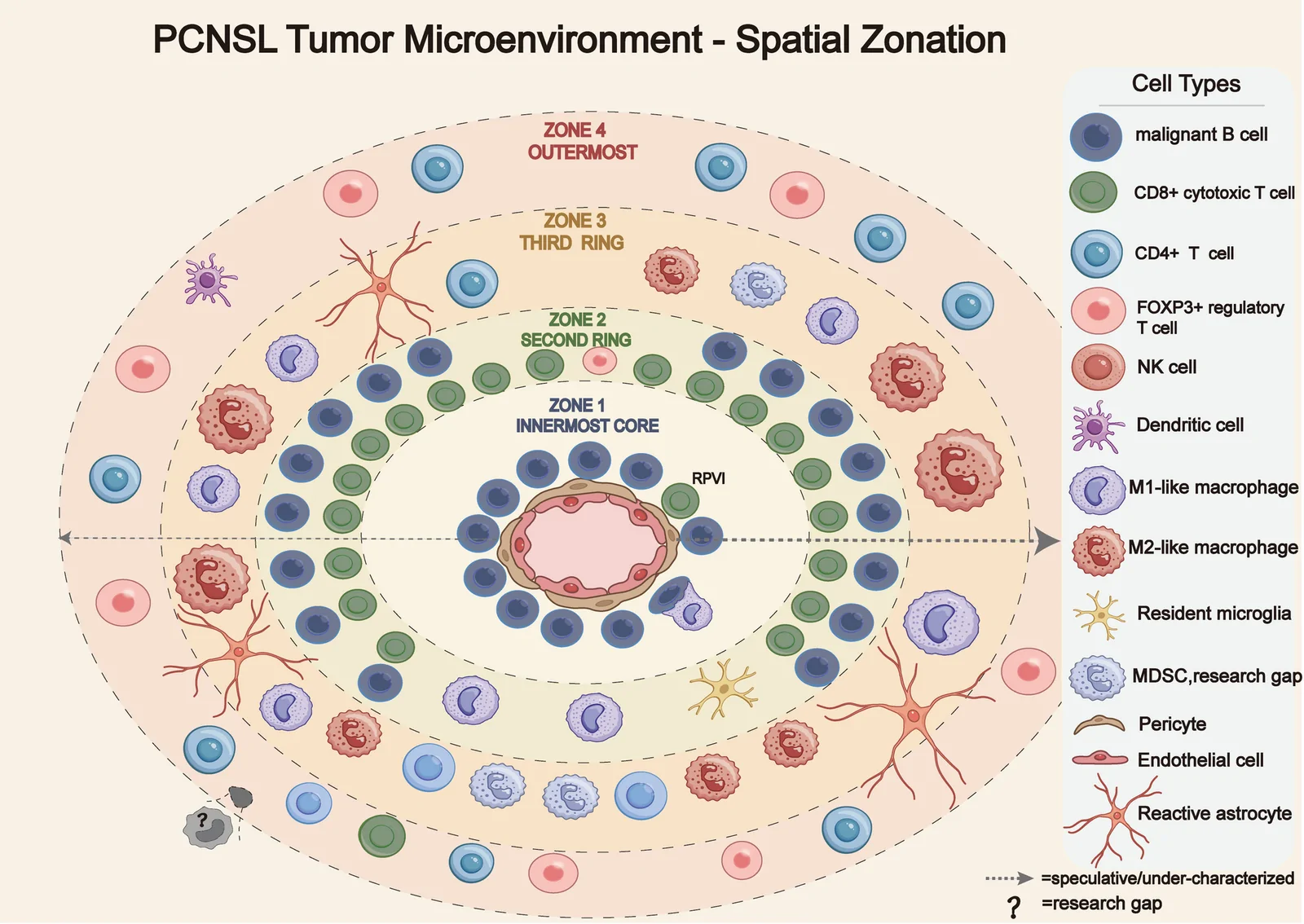
Spatial zonation of the tumor microenvironment in PCNSL. PCNSL features a highly structured four-layered ecosystem. Zone 1 (Core): Malignant B cells with reactive CD8⁺ T-cell infiltrates. Zone 2 (Intratumoral): Malignant B cells, CD8⁺ T cells and tumor-associated macrophages (TAMs). Zone 3 (Necrotic edge): Immunosuppressive shift dominated by M2-like macrophages and reactive astrogliosis. Zone 4 (Peritumoral): CD4⁺ T-cell enrichment, with sparse Tregs, NK, and DCs. This profound spatial heterogeneity blunts effective antitumor immunity. Uncharacterized populations (e.g., MDSCs) are marked with dashed lines/question marks. RPVI, reactive perivascular T-cell infiltrates

The PCNSL immune microenvironment is organized into four spatially distinct zones: perivascular, tumor parenchyma, peri-necrotic, and peritumoral brain parenchyma (Fig. 2). The perivascular zone harbors malignant B cells, reactive perivascular T-cell infiltrates (RPVI), and pericytes/perivascular mononuclear phagocytes [1, 29]. CD8⁺ T cells and TAMs - comprising monocyte-derived macrophages and microglia—predominate in the tumor parenchyma. M2-like macrophages accompanied by reactive gliosis are frequently observed at peri-necrotic borders. CD4⁺ T cells are relatively enriched in the peritumoral parenchyma, whereas FOXP3⁺ regulatory T cells (Tregs) are globally sparse [30, 31]. Natural killer (NK) cells and dendritic cells (DCs) are rarely or not systematically detected [32]. This architecture is consistent with a populated but functionally suppressed immune ecology. Myeloid-derived suppressor cells (MDSCs) have been discussed in the context of PCNSL-associated immunosuppression, yet in situ quantification and causal functional evidence remain incomplete [32]. Non-tumoral B cells, neutrophils, and mast cells, while recognized in the TME of extranodal lymphomas more broadly, lack systematic in situ characterization in PCNSL and represent important research gaps.

### Chemokine networks and tumor B-cell survival signals

Pericytes and perivascular macrophages secrete CXCL9 and CXCL12, recruiting CXCR3⁺CD8⁺ T cells to form RPVI [30]. Co-expression of CXCL9 and CXCL12 on tumor vasculature generates heterocomplexes that interact with CXCR4⁺ tumor B cells, maintaining their perivascular distribution [29]. Astrocytes contribute CXCL12, CCL2, CXCL1, and CXCL10 to the PCNSL chemokine network [32]. IL-4, produced autocrinally and paracrinally by endothelial cells and tumor B cells, promotes tumor cell proliferation and survival through downstream targets including Cathepsin D, Caspase-8, CFLAR, and XBP1 [1, 33]. Galectin-3 mediates endothelial-tumor BCR interactions, enhancing anti-apoptotic signaling and sustaining NF-κB activation [34]. AQP4 expressed by endothelial cells dynamically regulates BBB permeability, supporting the perivascular enrichment of tumor B cells [35]. Tumor B cells harboring *MYD88*^*L265P*^ and *CD79B* mutations co-localize BCR and TLR9 on the endosomal membrane, assembling a ligand-independent, sustained NF-κB/mTOR feedforward complex (My-T-BCR supercomplex) [36]. In parallel, tumor B cells receive dual survival support through BAFF (TNFSF13B) and APRIL (TNFSF13) signaling via BAFFR (TNFRSF13C), BCMA (TNFRSF17), and TACI (TNFRSF13B). Notably, CD20⁺ tumor cells co-express both ligands and all three receptors, establishing an autocrine circuit that sustains NF-κB activation [37–39]. This signaling network is further reinforced by astrocyte-derived BAFF, which provides additional paracrine support for NF-κB signaling within the CNS microenvironment [38].

### Myeloid suppressive axis

TAMs and microglia constitute the most abundant and consistently immunosuppressive populations within the PCNSL microenvironment, deriving from both resident microglial reprogramming and peripheral monocyte infiltration [1]. Three TAM subpopulations are recognized: M1-like (CD68⁺CD163ˡᵒʷ), M2-like (CD68⁺CD163ʰⁱ), and CD68⁻CD163ʰⁱ [30, 32, 40]. The immunosuppressive M2-like macrophage subset is significantly enriched in PCNSL compared with systemic DLBCL [41]. M2-like macrophages preferentially accumulate in necrosis-associated regions, where they produce immunosuppressive and pro-tumorigenic factors, including IL-10, TGF-β, VEGF, and matrix metalloproteinases, collectively orchestrating an immunosuppressive, pro-angiogenic, and matrix-remodeling tumor niche [1, 32, 40]. Receptor-mediated IL-10 signaling in PCNSL activates downstream STAT3, thereby sustaining malignant B-cell survival and proliferation [42, 43]. Consistently, cerebrospinal fluid (CSF) IL-10 levels correlate with both the extent of TAM infiltration and patient prognosis [44]. In parallel, M2-like macrophages and microglia express high levels of PD-L1 and Indoleamine 2,3-dioxygenase (IDO) [32, 45]. Through the tryptophan–kynurenine metabolic pathway, IDO1 depletes local tryptophan and accumulates immunosuppressive kynurenine metabolites, thereby impairing effector T cell and NK cell function [46, 47]. Recent spatial multi-omics studies have uncovered a TREM2⁺ lipid-associated macrophage subset in PCNSL, in which APOE-SORL1/LSR-dependent lipid trafficking drives metabolic reprogramming, whereas SPP1-CD44 signaling reinforces macrophage-T cell dysfunction, collectively contributing to an immunosuppressive tumor niche [41, 48].

### T-cell exhaustion and immune escape

Despite CD8⁺ cytotoxic T lymphocyte (CTL) infiltration, effector function in PCNSL is restrained by multiple overlapping mechanisms [1, 32]. These include (i) tumor cell-driven destabilization of HLA class I/II expression, resulting in defective antigen presentation; (ii) loss of CD58, the ligand for the T-cell co-stimulatory receptor CD2, which weakens CD2-mediated signaling and thereby impairs CTL recognition and activation [32, 49–51]; (iii) constitutive activation of the PD-1/PD-L1 axis suppresses CTL activation and cytotoxic function [32, 52, 53]; (iv) TIM-3 is highly expressed on tumor-infiltrating CD8⁺ T cells and marks an exhausted phenotype with impaired cytotoxicity, while its ligand Galectin-9, predominantly produced by M2-like macrophages, couples myeloid-derived suppression to T cell exhaustion within the tumor microenvironment [1, 54]; (v) TGF-β2, the predominant TGF-β isoform in the brain and produced by microenvironmental cells, suppresses pro-inflammatory programs and interferes with HLA class II upregulation, thereby impairing CTLs priming [55]; (vi) tumor cell-expressed Fas ligand (FasL) engages Fas (CD95) on cytotoxic T lymphocytes, triggering apoptosis of Fas-expressing effector T cells [56]; (vii) VEGF derived from TAMs, tumor cells, and Tregs reshapes the tumor microenvironment by fostering the recruitment and accumulation of MDSCs and Tregs, while concurrently restricting CTLs infiltration and antitumor immune activity [32, 57].

## The mutational landscape of PCNSL

### Somatic mutation landscape: an MCD/C5-like genomic program

PCNSL is dominated by an MCD/C5-like molecular program, with recurrent lesions converging on a limited set of oncogenic pathways [1]. Constitutive activation of the BCR-TLR-NF-κB axis is a defining molecular feature of PCNSL, driven predominantly by the frequent co-occurrence of *MYD88*^*L265P*^ (38%–94%, depending on cohort) and *CD79B*^*Y196*^ (45%–63%) mutations [58], both of which are substantially more prevalent than in systemic DLBCL (approximately 17% and 11%, respectively) [1, 12, 36, 59–62]. Additional gain-of-function or activating lesions in *CARD11* [63], *TBL1XR1* [64, 65] and *NFKBIZ* [66, 67], together with loss of *TNFAIP3* or *SLIT2* [1, 68], further reinforce constitutive NF-κB signaling. Epigenetic regulators are recurrently altered in PCNSL, with *KMT2D* representing the most frequently mutated (22%-49%) epigenetic modifier [9], while *EP3*00 is enriched in Asian cohorts and associated with adverse clinical outcomes [69], and *HIST1H1E* characteristically enriched in PCNSL CS3 cluster [2]. Immune evasion in PCNSL is driven by recurrent alterations in *HLA-A*, *HLA-B*, *HLA-C*, *B2M*, *CD58*, and *CIITA*, which collectively compromise MHC class I/II antigen presentation and immune recognition [2]. Alterations affecting apoptosis and cell-cycle regulation are common in PCNSL, including frequent mutations in *PIM1* [2, 70], recurrent biallelic deletion of *CDKN2A* [59], and *PRDM1* inactivation through mutation and 6q21 loss [71], whereas *TP53* mutations generally occur at a comparatively lower frequency than in systemic DLBCL [72]. Recurrent genetic lesions also affect the PI3K-PTEN-AKT, RTK-RAS-MAPK, and Wnt/β-catenin pathways [70, 73]. Together with pervasive aberrant somatic hypermutation targeting *PAX5*, *RhoH*, and other loci, as well as a striking bias toward *IGHV4-34* usage, these alterations collectively define a molecular landscape distinct from that of systemic DLBCL [12, 65, 74].

### Copy number alteration landscape: a parallel second driver layer

The genomic landscape of PCNSL is further shaped by recurrent copy number alterations (CNAs), which cluster at chromosomal hotspots including 19p13.13, 18q21.23, 12q15, 11q23.3, 6p21, 1q32.1, and 2q31.2, among others [2]. Additional recurrent copy number alterations include gains at 3q12.3, which upregulate NFKBIZ and potentiate NF-κB signaling, and gains at 9p24.1 encompassing *CD274 (PD-L1)*, *PDCD1LG2 (PD-L2)*, and *JAK2*, although their transcriptional consequences appear to vary across cohorts [12, 75]. Recurrent chromosomal deletions converge on pathways governing immune surveillance, cell-cycle control, and B-cell differentiation. Key events include loss of 6p21 (HLA class I/II loci), 9p21.3 (*CDKN2A/B*), and 6q21 (*TNFAIP3 and PRDM1*), the latter coupling constitutive NF-κB activation with impaired plasma cell differentiation. Additional recurrent losses affecting *B2M*, *CD58*, and *ETV6* further reinforce immune evasion and tumor progression [2]. Moreover, uniparental disomy and copy-neutral loss of heterozygosity amplify the impact of these driver alterations.

### Additional genomic and epigenomic determinants of PCNSL pathogenesis

Beyond the BCR-TLR-NF-κB axis, PCNSL biology is shaped by cell-cycle deregulation, differentiation arrest, immune escape, and epigenetic disturbance. CDKN2A/B loss represents a pivotal cell-cycle lesion in PCNSL. Deletion of p16INK4a dismantles CDK4/6-RB-mediated restriction of G1/S progression, while concomitant loss of p14ARF compromises the MDM2-p53 surveillance axis. Together, these alterations drive unchecked proliferative signaling and provide an alternative route to p53 pathway attenuation despite the relatively low prevalence of *TP53* mutations [76]. At the epigenetic level, recurrent alterations in *KMT2D*, *EP300*, and *HIST1H1E/H1-4* implicate chromatin dysregulation as a fundamental driver of the aberrant transcriptional programs that sustain PCNSL. Loss of KMT2D and EP300 is predicted to perturb enhancer architecture through disrupted H3K4 methylation and H3K27 acetylation, respectively, whereas histone H1 alterations may further reshape chromatin accessibility. However, these mechanistic models are largely extrapolated from systemic DLBCL and remain to be directly validated in the unique biological context of PCNSL. Upregulation of miR-17-5p, miR-155, miR-21, and miR-196b further highlights the contribution of non-coding RNA networks to PCNSL pathogenesis, linking post-transcriptional regulation to NF-κB activation, B-cell fitness, and therapeutic resistance. However, the regulatory circuitry through which these microRNAs coordinate malignant signaling programs remains incompletely resolved [77–81]. Recent transcriptomic and methylomic analyses support EBV-positive PCNSL as a biologically distinct molecular subtype characterized by virus-driven transcriptional programs, activation of IL-10-JAK/STAT and NOTCH signaling, engagement of viral life-cycle pathways, and widespread promoter hypomethylation accompanied by a unique epigenetic landscape. In contrast, EBV-negative tumors preferentially retain dependency on BCR and Wnt/β-catenin signaling, underscoring divergent pathogenic routes within PCNSL [82].

### Molecular stratification revealing intrinsic heterogeneity of PCNSL

**Fig. 3:**
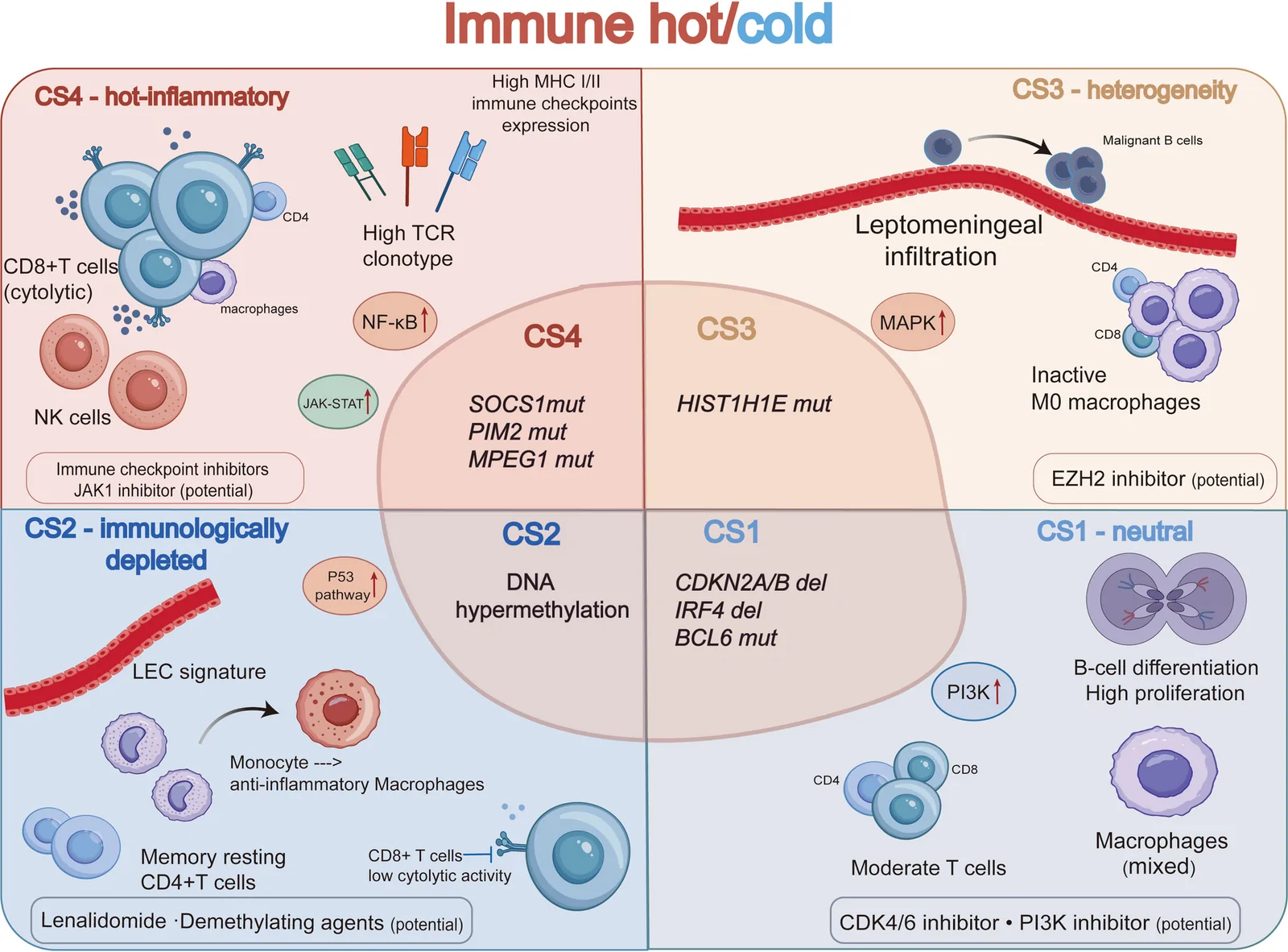
Molecular-immune subtypes of PCNSL define distinct tumor ecosystems and clinical outcomes. Multi-omics profiling stratifies PCNSL into four subtypes across an immune-cold to -hot spectrum. CS1 (bottom right): Immune-neutral, hyperproliferative state driven by *BCL6* mutation, *IRF4* and *CDKN2A/B* loss (Treatment regimen (potential): CDK4/6 plus PI3K inhibitors). CS2 (bottom left): Immunologically depleted niche governed by DNA hypermethylation (Treatment regimen (potential): lenalidomide or demethylating agents). CS3 (top right): Heterogeneous, *HIST1H1E*-mutated ecosystem dominated by inactive M0 macrophages (Treatment regimen (potential): EZH2 inhibitors). CS4 (top left): Immune-hot, *SOCS1/PIM2/MPEG1*-mutated subtype with high CD8⁺ T-cell infiltration and elevated immune checkpoints expression (Treatment regimen (potential): immune checkpoint or JAK1 inhibitors). This framework links genomic drivers directly to precision oncology. mut, mutation; del, deletion; LEC, lymphatic endothelial cell

**Fig. 4:**
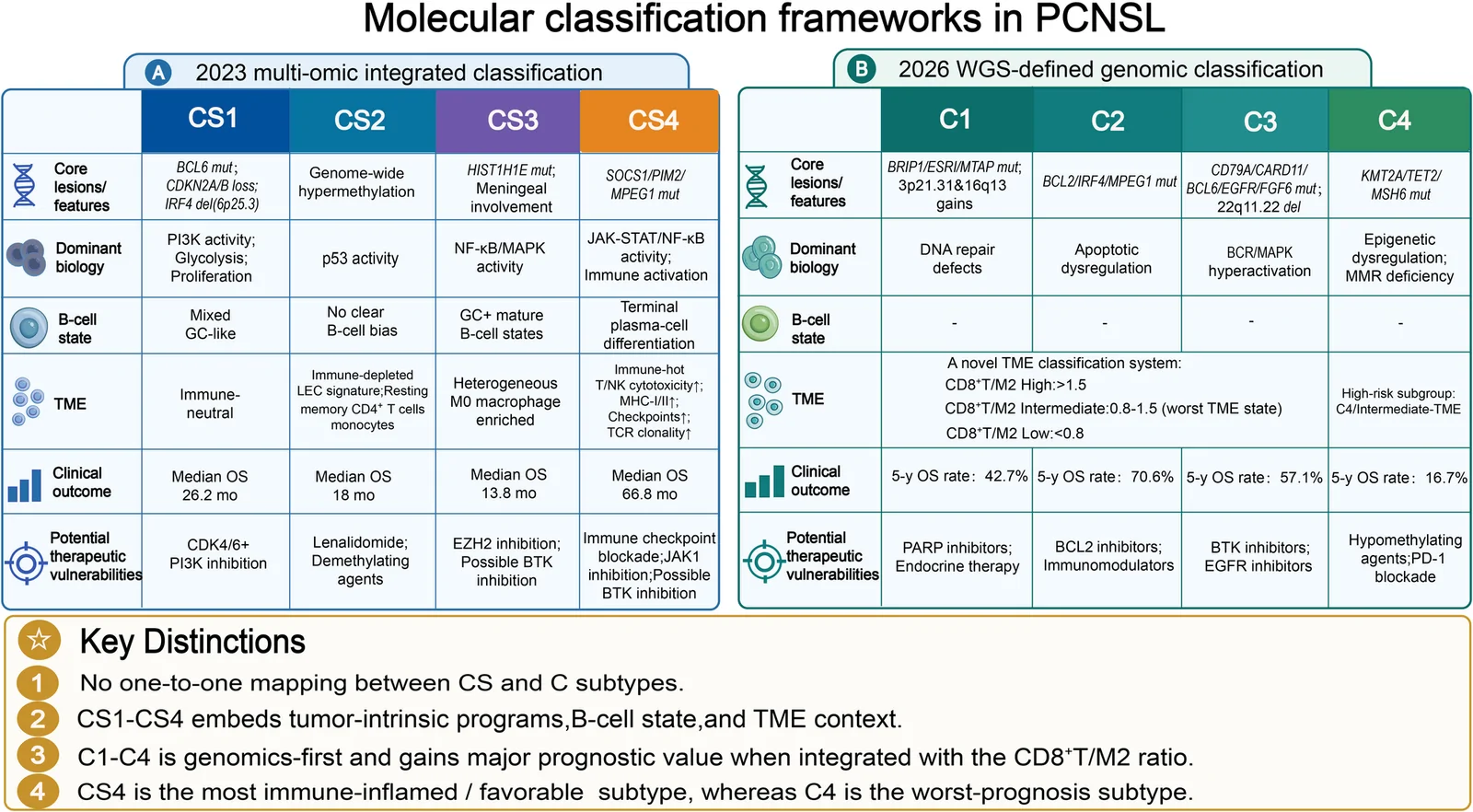
Comparison of molecular classification frameworks in PCNSL. The 2023 multi-omic integrated classification (A: CS1-CS4) is compared with the 2026 WGS-defined genomic classification (B: C1-C4). Subtype characteristics are summarized according to core lesions/features, dominant biology, B-cell state, tumor microenvironment, clinical outcome, and potential therapeutic vulnerabilities. WGS, whole-genome sequencing; mut, mutation; del, deletion; CD8⁺T, CD8-positive T cells; M2, M2-like macrophages; TCR, T-cell receptor; 5-y OS, 5-year overall survival; mo, months; LEC, lymphatic endothelial cell; TME, tumor microenvironment

Four molecular clusters, CS1-CS4, defined through integrative multi-omics profiling, delineate the major dimensions of PCNSL heterogeneity, encompassing distinct evolutionary drivers, B-cell differentiation states, tumor microenvironmental features, and clinical outcomes [2]. CS1 is characterized by *BCL6* mutations, *CDKN2A/B* loss, *IRF4* deletion (6p25.3), accompanied by high proliferative activity, a mixed germinal center-like transcriptional state. CS1 is defined by an immune-neutral tumor microenvironment, lacking both overt immune exclusion and active immune engagement. Clinically, it is associated with a median overall survival of 26.2 months and may reveal actionable therapeutic vulnerabilities to CDK4/6 or PI3K pathway inhibition. CS2 features genome-wide hypermethylation and an epigenetically enforced immune-exhausted state, accompanied by enrichment of endothelial cells, resting memory CD4⁺ T cells, and monocytes. Activation of γ-aminobutyric acid (GABA) biosynthetic programs may further reinforce this immunosuppressive milieu by dampening CD8⁺ T cell cytotoxicity and skewing monocytes toward an anti-inflammatory macrophage phenotype. Despite lacking a clear B cell differentiation bias, CS2 exhibits a prominent lymphatic endothelial cell signature and is clinically associated with a median overall survival of 18 months. CS3, the subtype with the poorest clinical outcome, is marked by recurrent *HIST1H1E* mutations, meningeal involvement, a heterogeneous immune landscape enriched for inactive M0 macrophages, and malignant B cells spanning germinal center and mature B cell states, potentially nominating EZH2 inhibition as a therapeutic entry point. CS4, an immune-inflamed *SOCS1/PIM2/MPEG1*-mutant subtype, integrates heightened immune checkpoint and MHC expression, JAK-STAT and NF-κB pathway activation, robust CD8⁺ T cell and NK cell cytotoxicity, increased TCR clonality, and terminal plasma cell differentiation, collectively aligning with the most favorable clinical outcome and nominating immune checkpoint blockade and JAK1 inhibition as rational therapeutic strategies [1, 2] (Fig. 3). The most recent molecular classification of PCNSL, based on whole-genome sequencing (WGS), defined four distinct genomic subtypes, designated C1-C4, each characterized by a unique constellation of driver alterations and dysregulated signaling pathways [3] (Fig. 4). C1 tumors are enriched for DNA repair pathway defects, with recurrent mutations in *BRIP1* and *MTAP* and copy number gains at 3p21.31 and 16q13. C2 tumors are driven by dysregulated apoptotic programs associated with *BCL2* and *IRF4* mutations. C3 tumors are defined by hyperactivation of BCR-MAPK signaling, frequently involving alterations in *CD79A*, *CARD11*, *BCL6*, and *EGFR*. In contrast, C4 tumors are characterized by epigenetic dysregulation and mismatch repair (MMR) deficiency, including mutations in *KMT2A*, *TET2*, and *MSH6*. Collectively, these molecular subtypes exhibit distinct clinical outcomes and therapeutic vulnerabilities, providing a clinically actionable framework for precision-targeted therapy in PCNSL [3].

## Contributions of lymphoma cell mutations to the TME

**Fig. 5:**
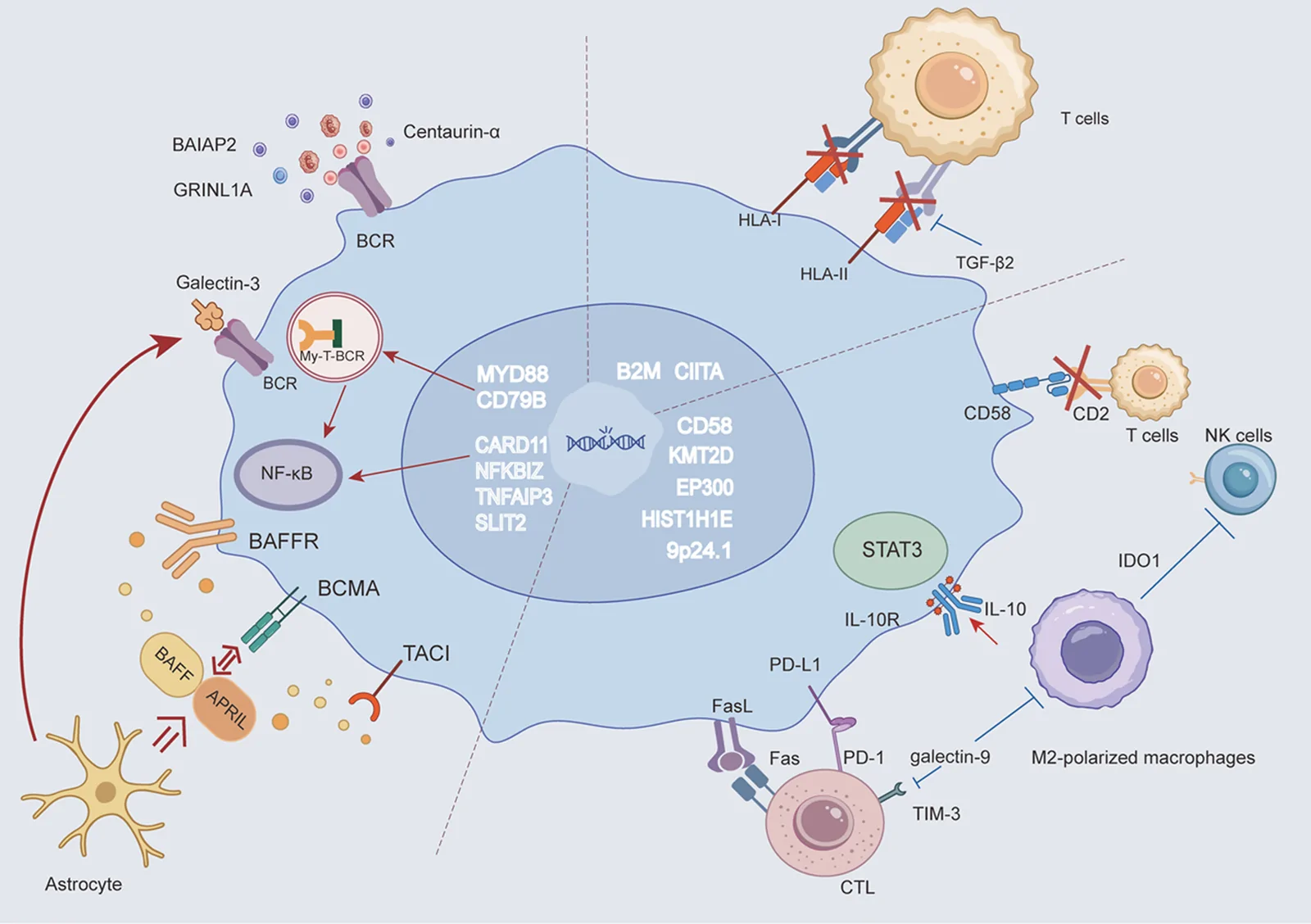
Genotype-driven mechanisms of tumor survival and immune escape in PCNSL. The lymphoma genotype acts as the central architect of the microenvironment across three sectors. Left: Chronic My-T-BCR/NF-κB activation, reinforced by genetic alterations, autoantigens, and astrocyte-derived BAFF/APRIL, sustains tumor cell survival. Top Right: Loss of *B2M* and *CIITA* disrupts antigen presentation and immune recognition, evading CD8⁺ T-cells. Bottom Right: CD58 loss, upregulated immune checkpoints - including PD-L1, FasL, and TIM-3-and IL-10/IDO1-mediated crosstalk with M2-like macrophages converge to drive profound T cell exhaustion. Together, these pathways establish an immune-permissive CNS niche that supports tumor persistence

The genetic alterations intrinsic to the PCNSL cells are not confined to driving cell proliferation; they function as the critical transcriptional nexus linking B-cell survival to extrinsic TME immunosuppression (Fig. 5).

### NF-κB pathway signaling as a TME modulator

Compared to systemic DLBCL, PCNSL harbors a unique repertoire of high-frequency genetic alterations that rewire the NF-κB signaling cascade via distinct modalities. Recurrent somatic mutations in *CD79B*, *MYD88* and *TBL1XR1* contribute to the constitutive activation of NF-kB [11, 64]. *CARD11* mutations, located in the coiled-coil region, which may activate NF-κB, were detected in 16% of PCNSL [63]. Recurrent *PRDM1* inactivation in PCNSL fuels NF-κB signaling by enhancing chromatin accessibility and promoting the transcription of its target genes [17]. Recurrent proliferation at 3q12.3, noted in 45% of PCNSL patients, drives the overexpression of *NFKBIZ*, which encodes the NF-κB coactivator IκB-ζ [83]. Constitutive activation of NF-κB, predominantly driven by these alterations, transforms the lymphoma cell into a factory of pro-tumorigenic signaling molecules. While the primary function of this cascade is the transcription of anti-apoptotic and pro-survival factors, such as BCL2 and potentially MYC, its indispensable secondary function is the sustained production of TME-modulating factors [84]. Chronic NF-κB activation induces a broad repertoire of pro-inflammatory cytokines, including TNF-α and IL-6, chemokines such as CXCL8, CXCL1, and CCL2, and adhesion molecules such as ICAM-1, collectively facilitating immune-cell recruitment. Within the tumor microenvironment, sustained NF-κB signaling can further skew recruited macrophages toward an immunosuppressive phenotype through upregulation of PD-L1 and arginase-1 [84]. NF-κB may also promote immunosuppression by increasing the levels of immunosuppressive cytokines (e.g., IL-10 and TGF-β) and recruiting MDSCs, thereby promoting tumor progression [84]. Consistent with this dual role, PCNSL subtypes with prominent pathway activation can exhibit features of an inflamed yet immunoregulatory microenvironment. Thus, while the primary function of NF-κB is to promote the survival of tumor cells, it also plays a vital secondary role in cultivating the immune microenvironment essential for chronic growth in the central nervous system.

### Genomic instability and immune checkpoint dysregulation

In PCNSL, mutations in *HLA-A*, *HLA-B*, and *HLA-C* [2], alongside DNA deletions at loci such as 6p21.32, 6q21, 8q12-12.2, 9p21.3, 3p14.2, 4q35.2, 10q23.21, and 12p13.2 [71], lead to impaired MHC class I expression. Concurrently, focal deletion of *B2M* at 15q21.1, observed in 21.21% of cases, and chromosomal rearrangements involving *CIITA* compromise MHC class I/II expression and thereby impair tumor antigen presentation [83]. In addition, approximately 27% of PCNSL cases harbor *CD58* genetic alterations, including missense, nonsense, and frameshift mutations, as well as copy-number alterations, predominantly deletions, which can impair CD58 function or result in loss of its expression [2]. These alterations consequently weaken T cell-mediated antitumor immune surveillance. While a 67% amplification of the 9p24.1 locus (*PD-L1/PD-L2*) in one study has been implicated as a pivotal mechanism of immune evasion in PCNSL [83, 85], discordant findings across independent cohorts reveal no significant copy number alterations at this site [59]. Substantial variability in PD-L1 expression detection was observed, largely influenced by cellular context and the scoring methodology employed. Total microenvironmental PD-1/PD-L1 positivity spans 90% of cases when integrating both malignant and immune cells [86]. However, isolating the analysis to tumor cell membranes drops the positivity rate to 10% (2/20) [86]. In another study, PD-L1 positivity was defined as membranous or cytoplasmic staining in ≥ 1% of cells, with PD-L1 expression detected in tumor cells in 37.5% of PCNSL cases (12/32) [87]. These findings further underscore the complex and dynamic interplay of PD-1/PD-L1 signaling between tumor and immune cell compartments. The role of PD-1/PD-L1 signaling is still under investigation.

### Genetic differences in immune-competent vs. immune-suppressed PCNSL

The genetic landscape can differ significantly based on the host’s immune status. Immunodeficient PCNSL represents less than 10% of PCNSL cases (< 0.1% of Non-Hodgkin Lymphoma (NHL)) [88]. These patients are generally younger than those with non-immunosuppressed PCNSL and experience poor outcomes [89]. The genetic features of immunocompetent PCNSL significantly differ from those of immunosuppressed PCNSL. In immunosuppressed cases, such as Epstein-Barr virus (EBV) (+) PCNSL, mutations in the BCR-dependent NF-κB pathway, copy number loss in the cell cycle and adhesion pathways, alterations in HLA class I/II, and mutations or deletions in the antigen presentation and processing pathways are rare compared to immunocompetent cases [88, 90]. EBV-positive PCNSL is characterized by an absence of *MYD88*, *CD79B*, and *PIM1* mutations, and is rarely classified as an ABC subtype [88]. Its intact HLA-I/II expression suggests that the antigen presentation pathway remains largely unaffected [88]. Nevertheless, EBV-positive PCNSL is associated with a tolerogenic TME, which may promote tumor escape through mechanisms of viral regulation [90]. The EBV infection is implicated in chronic NF-κB activation and immune evasion, enabling the tumor to bypass selective pressure related to *MYD88* and *CD79B* alterations. Elevated levels of macrophage markers CD68 and CD163, along with immune checkpoints, have been observed in EBV-positive, human immunodeficiency virus (HIV)-negative PCNSL cases [88]. Conversely, in acquired immune deficiency syndrome (AIDS)-related PCNSL, tumor cell immune escape is partly facilitated by a reduction in CD4 + T cells [91]. Additionally, the activation of the NF-κB p65/PD-L1 signaling pathway is linked to external cytokine stimulation, particularly tumor necrosis factor-α (TNF-α) and interferon-γ (IFN-γ), rather than being predominantly driven by *MYD88* and *CD79B* mutations [92]. This mechanistic divergence underscores the importance of stratifying patients based on immune status and the associated viral etiology.

## Contribution of genotypic variants and alterations on non-tumor cells in TME

While the lymphoma cell initiates the immunosuppressive signal, the host’s resident cells, particularly microglia and associated neuroglial components, are the necessary effectors that translate this signal into functional immune suppression.

### Intrinsic immune cell and host factors influencing CNS immunity

The TME features a continuous, two-way interaction between lymphoma cells and non-cancerous elements, including endothelial cells, fibroblasts, and the extracellular matrix. The specific physiology of cells residing in the CNS influences how they respond to chemokines released by lymphoma. As a key resident immune population within the CNS, microglia are capable of infiltrating tumors and act as primary mononuclear phagocytes, contributing uniquely to the TME. Other non-malignant immune cell types that modulate immune responses include TAMs and TILs.

### Molecular switches governing microglia/TAM polarization

The TME of PCNSL exhibits significant infiltration of TAMs, primarily originating from CNS-resident microglia [41, 93]. TAMs are pivotal within the TME of PCNSL, notably affecting tumor growth, proliferation and anti-tumor immune response [94]. The microglial phenotype is shaped by the cytokine environment produced by lymphoma or stromal cells, leading to the generation of both pro-inflammatory M1 and anti-inflammatory M2-like macrophages [57]. Among these, M2-like macrophages, which possess immunosuppressive functions, can be activated by cytokines such as IL-10, IL-4, IL-13, and M-CSF [32, 95]. A study focused on local copy number alterations identified amplification at 1q32.1 (33%, IL-10), which may influence TAM polarization by modulating IL-10 expression and secretion [2, 5]. *MYD88/CD79B* mutation-induced activation of the NF-κB signaling pathway facilitates the release of IL-10, CCL2, M-CSF and other cytokines, promoting M2 recruitment and polarization [36, 96, 97]. M2-like macrophages reinforce an immunosuppressive microenvironment through the production of anti-inflammatory mediators, including IL-10 and TGF-β, thereby suppressing CTLs activity, promoting Tregs expansion, and impairing NK cells function via altered antigen presentation, ultimately facilitating tumor immune evasion [98]. Concurrently, STAT3 activation serves as a molecular switch that regulates the polarization of macrophages and microglia toward an immunosuppressive M2 phenotype. In PCNSL cells, STAT3 activation correlates significantly with the number of M2-like macrophages [99], and the level of STAT3 activation in *MYD88*^*L265P*^ mutant lymphomas is notably higher than in *MYD88* wild-type lymphomas [100]. This causal relationship - spanning genetic variation in lymphoma cells, activation of downstream signaling pathways, cytokine release, and M2 polarization - underpins the identification of the genetic-TME axis in PCNSL.

### Molecular abnormalities disrupting cytotoxic T lymphocyte (CTL) immune surveillance

The genomic landscape of PCNSL is defined by recurrent alterations that effectively neutralize the adaptive immune response, leading to the functional paralysis and physical exclusion of CD8^+^ CTLs. It was demonstrated that the expression of granzyme B and perforin in CD8^+^ T cells of *MYD88*^*L265P*^ mutant lymphoma patients was significantly lower than that of *MYD88* wild-type (WT) lymphoma patients, suggesting that the *MYD88* mutation would be detrimental to the killing function of CD8^+^ T cells [100]. The antigen-presentation and co-stimulatory abnormalities described above, particularly those involving *HLA/B2M/CIITA* and *CD58*, further compromise tumor immune recognition. Rather than acting as discrete mechanisms, these defects converge functionally: impaired peptide-MHC presentation limits TCR engagement, while attenuated co-stimulatory signaling and reduced effector competence collectively blunt CTL activity [12, 101, 102]. Consequently, infiltrating CTLs may remain physically present yet functionally compromised, establishing a multilayered barrier to adaptive immune surveillance within the CNS niche.

### Non-canonical signaling in the CNS niche: receptor-ligand crosstalk

The distinct phenomenon of angiotropism, in which lymphoma cells cluster around blood vessels, underscores a significant level of interaction with endothelial cells [33, 103]. This crosstalk is mediated by secreted factors such as VEGF and various chemokines released by both M2-like macrophages and the lymphoma cells themselves [104]. This communication is thought to promote local angiogenesis, ensure a sufficient nutrient supply, and disrupt the BBB, all contributing to tumor growth and infiltration.

## Co-evolution of the crosstalk of cancer and LME cells during progression

The TME is a dynamic and evolving ecosystem that is not merely a passive bystander but an active participant in tumor progression, therapy response, and invasive potential [105]. In PCNSL, the crosstalk between the malignant B-cells and the local immune microenvironment (LME) is co-evolutionary, creating a stable, self-reinforcing loop that drives disease progression. A co-evolution model treats PCNSL not as a tumor mass with a passive infiltrate, but as a dynamically coupled system: malignant B cells modify the brain’s innate immune and stromal programs, while brain-resident cells (microglia, astrocytes, endothelial cells) impose selection pressures that favor tumor states best adapted to (i) immune restriction, (ii) compartmentalized trafficking (CSF/perivascular space), and (iii) nutrient/oxygen constraints.

### The dynamic feedback loop: lymphoma genetics driving microenvironmental contextual cellular responses

PCNSL evolves within a uniquely constrained immune niche, where malignant B cells and LME cells engage in reciprocal and adaptive signaling. Rather than passively inhabiting the CNS, lymphoma cells actively instruct the surrounding stroma through genetically encoded programs. As described above, constitutive activation of NF-κB signaling, driven by recurrent genetic lesions, represents a central organizing axis of tumor-microenvironment crosstalk in PCNSL [69, 106]. Beyond promoting tumor-cell survival, NF-κB signaling induces the secretion of cytokines and chemokines that recruit and shape immune populations, including TAMs. These TAMs, in turn, produce TGF-β and additional IL-10, further suppressing cytotoxic T-cell activity, promoting angiogenesis, and facilitating tumor progression [84]. In particular, TAM-derived suppressive cues can sustain STAT3-centered signaling in malignant B cells, creating a feed-forward circuit that stabilizes tumor-promoting inflammation [107]. Recurrent genomic alterations further remodel contextual cellular responses, while non-malignant cells function not as passive bystanders but as adaptive partners that shield tumor clones from immune pressure. This reciprocal reinforcement converts genetically initiated signaling into a self-sustaining ecological program, enabling lymphoma cells and the CNS stroma to co-adapt and maintain a permissive niche for persistent tumor growth.

### Hypoxia, metabolic reprogramming, and TME toxicity

The CNS microenvironment imposes metabolic constraints that further shape tumor–stroma co-evolution. PCNSL lesions frequently exhibit perivascular growth patterns, yet aberrant vascular remodeling and high proliferative indices generate hypoxic niches. Hypoxia-induced endoplasmic reticulum stress (ERS) and unfolded protein response (UPR) might be associated with aggregative perivascular tumor cells (APVTs) formation in PCNSL and its poor clinical outcomes [108]. Meanwhile, PCNSL differs significantly from other brain tumors in terms of hypoxia-inducible factor 1α (HIF-1α) signaling, and its hypoxic microenvironment is critical for PCNSL progression through activation of angiogenesis, immunosuppression, and metabolic reprogramming [109, 110]. Abnormal glucose metabolism mediated by HIF-1α played an essential role in the development of PCNSL [111]. HIF-1α stabilization promotes glycolytic reprogramming, lactate production, and VEGF-mediated angiogenesis, thereby coupling metabolic adaptation to stromal remodeling [112, 113]. Lactate accumulation acidifies the local milieu and impairs the function of non-malignant immune cells, such as TILs, while promoting M2-like polarization of TAMs - a metabolic symbiosis that reinforces immune tolerance [114–116]. Constitutive activation of STAT3 signaling promotes glycolysis through the transcriptional activation of hypoxia-inducible factors, thereby altering mitochondrial activity and linking oncogenic signaling to bioenergetic rewiring [117]. Moreover, PI3K/AKT/mTOR activation - reported in subsets of PCNSL - supports anabolic growth and resistance to metabolic stress [118]. These pathways not only sustain tumor cell fitness but also alter nutrient competition within the LME, depriving T cells of glucose and amino acids essential for cytotoxic activity.

Therapeutically, this metabolic plasticity may underlie partial responses to high-dose methotrexate (HD-MTX) and resistance to targeted agents [119, 120]. Emerging strategies targeting BTK, mTOR, or lactate metabolism may disrupt this metabolic crosstalk, potentially restoring immune competence within the CNS niche. Thus, metabolic reprogramming is not merely an intrinsic hallmark of PCNSL cells but a cooperative adaptation that conditions TME toxicity and immune dysfunction.

### TME heterogeneity and clonal selection during relapse and therapy resistance

Relapsed PCNSL illustrates the evolutionary consequences of sustained tumor–microenvironment interplay. Genomic analyses reveal clonal diversification under therapeutic pressure, often retaining trunk mutations (e.g., *MYD88*^L265P^, *TBL1XR1*, *BCL6* translocations) while acquiring additional immune escape or proliferative lesions [121]. Immune evasion is generally acquired during the later stages of tumor evolution and likely plays a critical role in tumor establishment after malignant cells colonize the immune-privileged niche of the CNS. One study has proposed that PCNSL relapse may arise from a persistent, relatively chemoresistant common progenitor cell (CPC) reservoir rather than solely from residual tumor cells surviving first-line treatment. Although this model offers a potential explanation for the evolutionary origin of recurrent disease, it remains to be validated in independent cohorts. If confirmed, the persistence of dormant CPCs could have implications for consolidation and maintenance strategies aimed at achieving more durable disease control, including the exploration of targeted approaches such as BTK or HDAC3 inhibition [121].

Spatial heterogeneity within the CNS - across parenchymal, perivascular, and leptomeningeal compartments - creates regionally distinct immune ecologies that differentially constrain clonal outgrowth. Single-cell and spatial transcriptomic studies in CNS lymphomas have begun to uncover heterogeneous malignant B cell clusters, diverse TAM states and variable T cell exhaustion signatures, suggesting that immune pressure is neither uniform nor static [122]. Tumor cells may adopt an “immune pressure-sensing model” that drives distinct modes of TME remodeling, promoting the formation of either barrier-like or immune-cold microenvironments in response to the intensity and nature of immune selective pressure [123]. Although immune checkpoint blockade (PD-1, CTLA-4, TIM-3) has shown activity in relapsed disease, responses remain heterogeneous, likely reflecting pre-existing diversity in antigen presentation capacity, T cell infiltration, and myeloid composition [102, 122].

Chemotherapy itself remodels the LME. HD-MTX can transiently deplete immune cells and alter cytokine gradients, potentially selecting for clones adapted to low-immunity states [124–126]. Moreover, therapy-induced senescence or metabolic stress in stromal cells may generate secondary inflammatory niches that support resistant subclones. Loss of HLA molecules or CD58 expression further impairs immune-mediated clearance at relapse, as well as the efficacy of immunotherapies, such as PD1 inhibitors or CAR-T therapies [101, 127–130]. Collectively, these observations support a model in which PCNSL progression is not linear but eco-evolutionary: genetic diversification and microenvironmental selection coalesce to produce therapy-resistant ecosystems.

## Targeted therapy for PCNSL TME

The therapeutic landscape for PCNSL is shifting toward strategies that modulate the TME, particularly in the relapsed/refractory (R/R) setting. These approaches aim to dismantle the immune-privileged and immunosuppressive state of the CNS to restore anti-tumor vigilance (Table 1).

### Immune checkpoint blockade and cellular immunotherapy

The PD-1/PD-L1 axis remains a therapeutic target for overcoming TME-mediated immune evasion in PCNSL. Clinical studies of nivolumab and pembrolizumab monotherapy in relapsed or refractory PCNSL, including NCT02857426 and NCT02779101, have reported clinically relevant outcomes (Table 1) [131, 132]. In one multicenter phase II study, PD-1 blockade achieved an overall response rate (ORR) of 26% with an acceptable toxicity profile, indicating modest single-agent efficacy [132]. These findings support further evaluation of PD-1 blockade within rational combination strategies. Current research suggests that the efficacy of these agents is hindered by a ‘cold’ TME characterized by low T-cell infiltration. Consequently, the focus has shifted toward CAR T-cell therapy. Preliminary clinical data indicate that CD19-targeted CAR T-cells can effectively cross the BBB [133–135], achieving durable remissions by inducing a robust local inflammatory response that overcomes the intrinsic immune-inhibitory signals of the CNS. CAR T-cell therapy may provide a feasible and potentially curative option for R/R PCNSL patients who fail conventional chemotherapy [136–141]. Although neurotoxicity may be somewhat more frequent or severe than that observed in systemic DLBCL, available evidence suggests that these events are generally manageable with appropriate monitoring and supportive care [139]. Accordingly, cytokine release syndrome (CRS) and immune effector cell-associated neurotoxicity syndrome (ICANS) remain important safety considerations but do not appear to constitute an absolute barrier to the use of CAR T-cell therapy in appropriately selected patients with PCNSL.

### Reprogramming the TME compartment: BTK and XPO1 inhibition

Emerging evidence indicates that Bruton’s tyrosine kinase (BTK) inhibitors, such as ibrutinib, exert both cell-intrinsic and microenvironmental effects, directly promoting apoptosis in malignant B cells and concurrently reshaping the tumor microenvironment [142]. Studies have shown that ibrutinib can interfere with tumor cell access to pro-proliferative microenvironmental cues, promotes CD4⁺ T cell differentiation toward Th1 helper programs, and enhances antitumor immune surveillance [143, 144]. In addition, a novel oral BTK inhibitor, HZ-A-018, has shown potent antitumor activity and robust BBB penetration. In a multicenter phase I trial, HZ-A-018 monotherapy was well tolerated in patients with R/R PCNSL and elicited a substantially improved response rate. At the recommended phase II dose (RP2D), HZ-A-018 achieved an ORR of 72.7% and a 12-month overall survival rate of 90.5%, with grade ≥ 3 adverse events occurring in 19.2% of patients. Nevertheless, further clinical validation in larger cohorts is warranted [145].

Meanwhile, BTK inhibitor-based synergistic strategies continue to emerge, exemplified by combinations of BTK inhibitors with XPO1 (exportin 1) inhibitors. Mechanistically, the synergistic combination of BTK and XPO1 inhibitors (e.g., Selinexor) exploits innate immune responses by shifting the M1/M2 ratio. BTK signaling is essential for the survival and pro-tumorigenic functions of M2-like macrophages; its inhibition, particularly when combined with XPO1 blockade, leads to the downregulation of PD-1 and SIRPα (Signal Regulatory Protein Alpha) on these cells [146]. SIRPα is a critical “don’t eat me” signal that interacts with CD47 on tumor cells; its downregulation relieves the inhibition of phagocytosis, allowing TAMs to regain their pro-inflammatory M1-like characteristics and actively engulf malignant cells [147].

### STAT3 signaling and immunomodulation

The STAT3 signaling pathway serves as a pivotal node in maintaining an immunosuppressive TME. STAT3 undergoes constitutive activation via autocrine mechanisms, subsequently upregulating the expression of PD-L1 and IDO in both tumor cells and TAMs [99]. STAT3 inhibitors have demonstrated the ability to abrogate this expression, thereby stripping the tumor of its biochemical shield against T-cells [46].

### Synergistic multi-targeted regimens

Third-generation immunomodulatory drugs (IMiDs), such as pomalidomide and lenalidomide, have shown promise as maintenance therapies in relapsed or refractory patients [148]. Notably, their integration into combination regimens supports a therapeutic paradigm centered on the simultaneous targeting of multiple tumor-intrinsic and microenvironmental pathways. While IMiDs enhance T-cell and NK-cell activity, their combination with BTK inhibitors addresses the complex interplay between genomic drivers and the supportive TME, offering a more comprehensive strategy for managing R/R PCNSL. Clinical trials utilizing the R2I regimen (Rituximab, Lenalidomide, and Ibrutinib) capitalize on these multi-faceted interactions - simultaneously targeting the malignant B-cells, enhancing antibody-dependent cellular cytotoxicity (ADCC) via Rituximab, and stimulating the underlying immune architecture of the CNS [149].

**Table 1:** Targeted therapies for PCNSL tumor microenvironment

Regimen	Patient	Study	Study type	Number of patients	Key survival outcomes	References	BBB Penetration	Primary Mechanism of Action
PD-1/PD-L1 Blockade (Nivolumab, Pembrolizumab)	Relapsed or refractory PCNSL	NCT02857426	Case report	4	CR 3, PR 1, PFS 13–17^+^ months	Nayak et al., Blood (2017) [131]	To be further clarified.	Blocks PD-1/PD-L1 axis; reverses T-cell exhaustion in a ‘cold’ TME.
		NCT02779101	Phase II	50	ORR 26% (13/50, 8 CR + 5 PR)	Hoang-Xuan K et al., Blood (2020) [132]		
CD19 CAR T-cells	Relapsed or refractory CNSL	-	Retrospective (EBMT database)	100	2-year PFS: 28% 2-year OS: 37%	Ossami Saidy et al., Hemasphere (2025) [140]	High; active cellular trafficking across the BBB.	Direct tumor killing; induces local CNS inflammatory response to overcome immunosuppression.
		-	Retrospective (LOC network)	25	CR 16 (64%), PR 4 (16%)	Choquet, S et al., Am J Hematol 2024 [139]		
		NCT02445248	Phase I/II	12	CR 6 (50%)	Frigault et al., Blood (2022) [136]		
ASCT + CD19/22 CAR-T cells	Relapsed or refractory PCNSL	-	Retrospective	29	ORR 82.75%, CRR 72.41%	Zhou, X et al., Exp Hematol Oncol (2024) [141]		
BTK inhibitors	Relapsed or refractory PCNSL	jRCT2080223590	Phase I/II	44	ORR 63.6%	Hajime Yonezawa et al., Neuro-Oncology Advances (2024) [150]	High	Attack the survival of malignant B-cells and increase antitumor immune reaction (BTK protein is required for the tumor-promoting effect of macrophages)
	Relapsed or refractory CNSL	ChiCTR2400091821	Phase I	26	ORR 72.7%	Shenglan Li et al., Acta Pharmaceutica Sinica B (2026) [145]		
BTK + XPO1 Inhibition (Ibrutinib + Selinexor)	-	-	Preclinical	-	-	Jimenez I et al., J. Neurooncol (2020) [146]	High; both agents are brain-penetrant small molecules.	TAM reprogramming (M2 to M1); downregulates PD-1 and SIRPα/CD47 ‘don’t eat me’ signal.
STAT3 Inhibitors (e.g., WP1066)	-	-	Preclinical	-	-	Miyasato Y et al., J Clin Exp Hematop (2018) [46]	Moderate to High; CNS-optimized small molecules.	Abrogates STAT3 signaling to reduce PD-L1 and IDO expression in tumor cells and TAMs.
Lenalidomide	Relapsed or refractory PCNSL	NCT01542918	Phase I	14	ORR 64%	Rubenstein, J. L et al., Blood Adv (2018) [148]	Good	Modulates tumor microenvironment, enhances anti-tumor activity of T/ NK cells
Pomalidomide	Relapsed or refractory PCNSL	NCT01722305	Phase I	25	ORR 48%	Tun, H. W et al., Blood (2018) [151]	Good	
R2I Regimen (Rituximab, Lenalidomide, Ibrutinib)	Relapsed or refractory PCNSL	NCT03703167	Phase Ib	18	ORR 89% (16/18, 8 CR + 8 PR)	Schaff, L. R et al., Neuro Oncol (2025) [149]	Good for Ibr/Len; Rituximab penetration is variable but effective.	Synergistically targets B cells and activates T/NK cells; enhances ADCC.

## Conclusions

Collectively, recent advances have substantially refined our understanding of the molecular architecture and TME of PCNSL, revealing a tightly interconnected genetic-TME axis that underpins disease pathogenesis and therapeutic vulnerability. PCNSL emerges as a genetically defined variant of DLBCL, characterized by near-universal activation of NF-κB signaling driven by recurrent mutations in *MYD88*, *CD79B*, *PIM1*, and related genes, which sustain malignant B cells in a persistently activated, germinal center-experienced state. In parallel, defects in antigen presentation (e.g., HLA, B2M), upregulation of immune checkpoint pathways (e.g., PD-L1, TIM-3), and enrichment of immunosuppressive M2-like macrophages collectively establish a permissive immune milieu that facilitates immune evasion within the CNS.

These mechanistic insights are increasingly translating into therapeutic innovation. While high-dose methotrexate remains the clinical backbone, emerging strategies are redefining treatment paradigms: BTK inhibitors exploit chronic BCR/NF-κB dependency, immune checkpoint blockade restores antitumor immunity, and CD19-directed CAR-T cells demonstrate substantial efficacy in refractory disease. Complementary approaches - including immunomodulatory agents and combinatorial targeting of IL-10-driven STAT3 and metabolic programs, as well as aberrant PI3K/mTOR signaling - further expand the therapeutic landscape. Together, these advances highlight a paradigm shift from empiric cytotoxic therapy toward mechanism-based, precision oncology approaches, positioning the genetic-TME axis as both a conceptual framework and a therapeutic roadmap for the future management of PCNSL.

## Future directions

Advancing PCNSL toward true precision oncology will depend on the convergence of mechanistic biology, innovative trial design, and real-time molecular monitoring, with the goal of transforming a historically aggressive malignancy into a tractable, stratified disease defined by its genetic-TME circuitry. Future research will further favor the accelerated use of single-cell and spatial analyses, longitudinal CNS sampling, and integrated multi-omics to address intratumoral heterogeneity, clonal evolution, and niche adaptation. Advanced preclinical models (orthotopic PDX, organoids) that recapitulate the BBB and immune milieu will be crucial.

On the clinical front, biomarker-guided combination strategies should be prioritized, including rational integration of high-dose methotrexate with BTK inhibitors or PD-1 blockade, as well as combinatorial regimens such as BTK inhibition with immunomodulatory agents. Parallel efforts targeting the tumor microenvironment are likely to further expand therapeutic opportunities. However, key challenges - including limited drug penetration across the BBB, CNS immune privilege, and profound microenvironmental heterogeneity - must be systematically addressed to enable effective translation. Ultimately, exploiting the unique convergence of genetic drivers and niche-specific biology in PCNSL is expected to uncover novel, targetable dependencies - including My-T-BCR signaling, IL-10-STAT3-mediated immunometabolic circuits, and HIF1α-driven adaptation - thereby opening new therapeutic avenues for a historically intractable malignancy. PCNSL provides a unique model to interrogate how immune privilege, spatial confinement, and limited drug penetrance shape cancer evolution and therapeutic response. Dissecting these principles may not only redefine precision medicine for PCNSL but also yield generalizable insights into cancers arising within specialized tissue niches.

## Data Availability

No datasets were generated or analysed during the current study.

## Abbreviations

PCNSL: Primary central nervous system lymphoma
CNS: Central nervous system
TME: Tumor microenvironment
DLBCL: Diffuse large B-cell lymphoma
non-GCB: non-germinal center B-cell-like
BBB: Blood-brain barrier
ECM: Extracellular matrix
TILs: Tumor-infiltrating lymphocytes
TAMs: Tumor-associated macrophages
GEP: Gene expression profiling
GCB: Germinal center B cell-like
ABC: Activated B cell-like
IHC: Immunohistochemistry
IGHV: Immunoglobulin heavy-chain variable region
SHM: Somatic hypermutation
GC: Germinal center
CLL: Chronic lymphocytic leukemia
BcR IGs: B-cell receptor immunoglobulins
CNAs: Copy number alterations
RPVI: Reactive perivascular T-cell infiltrates
Tregs: Regulatory T cells
NK cells: Natural killer cells
DCs: Dendritic cells
MDSCs: Myeloid-derived suppressor cells
CSF: Cerebrospinal fluid
IDO: Indoleamine 2,3-dioxygenase
CTL: Cytotoxic T lymphocyte
FasL: Fas ligand
GABA: γ-aminobutyric acid
NHL: Non-Hodgkin Lymphoma
EBV: Epstein-Barr virus
HIV: Human immunodeficiency virus
AIDS: Acquired immune deficiency syndrome
TNF-α: Tumor necrosis factor-α
IFN-γ: Interferon-γ
WT: Wild-type
TCR: T Cell Receptor
LME: Local immune microenvironment
ERS: Endoplasmic reticulum stress
UPR: Unfolded protein response
APVTs: Aggregative perivascular tumor cells
HIF-1α: Hypoxia-inducible factor 1α
HD-MTX: High-dose methotrexate
CPC: Common progenitor cell
R/R: Relapsed/refractory
BTK: Bruton’s tyrosine kinase
XPO1: Exportin 1
SIRPα: Signal Regulatory Protein Alpha
IMiDs: Immunomodulatory drugs
ADCC: Antibody-dependent cellular cytotoxicity
LEC: Lymphatic endothelial cell
ORR: Overall response rate
CRS: Cytokine release syndrome
ICANS: Immune effector cell-associated neurotoxicity syndrome
RP2D: Recommended phase II dose

## References

[1] Ferreri AJM, Calimeri T, Cwynarski K, Dietrich J, Grommes C, Hoang-Xuan K, et al. Primary central nervous system lymphoma. Nat Rev Dis Primers. 2023;9(1):29. 10.1038/s41572-023-00439-0. PMC10637780 37322012

[2] Hernández-Verdin I, Kirasic E, Wienand K, Mokhtari K, Eimer S, Loiseau H, et al. Molecular and clinical diversity in primary central nervous system lymphoma. Annals oncology: official J Eur Soc Med Oncol. 2023;34(2):186–99. 10.1016/j.annonc.2022.11.002. 36402300

[3] Yuan X, Luo Q, Huang Y, Li C, Yu T, Guo S, et al. Integrated Genomic and Tumor Microenvironment Subtyping Improved Risk Stratification in Primary Central Nervous System Lymphoma. Am J Hematol. 2026. 10.1002/ajh.70396. PMC13544481 42252624

[4] Alaggio R, Amador C, Anagnostopoulos I, Attygalle AD, Araujo IBO, Berti E, et al. The 5th edition of the World Health Organization Classification of Haematolymphoid Tumours: Lymphoid Neoplasms. Leukemia. 2022;36(7):1720–48. 10.1038/s41375-022-01620-2. PMC9214472 35732829

[5] Lorenzen S, Zeremski V, Berisha M, Mougiakakos D, Haage TR. Unraveling the immune microenvironment in primary CNS lymphoma. Biomark Res. 2026;14(1). 10.1186/s40364-026-00945-9. PMC13227751 42226261

[6] Dabrowska-Iwanicka A, Nowakowski GS. DLBCL: who is high risk and how should treatment be optimized? Blood. 2024;144(25):2573–82. 10.1182/blood.2023020779. 37922443

[7] Camilleri-Broët S, Crinière E, Broët P, Delwail V, Mokhtari K, Moreau A, et al. A uniform activated B-cell-like immunophenotype might explain the poor prognosis of primary central nervous system lymphomas: analysis of 83 cases. Blood. 2006;107(1):190–6. 10.1182/blood-2005-03-1024. 16150948

[8] Roschewski M, Hodson DJ. Diffuse large B-cell lymphoma involving the central nervous system: biologic rationale for targeted therapy. Haematologica. 2024;109(2):388–400. 10.3324/haematol.2021.278613. PMC10828633 37706315

[9] Bödör C, Alpár D, Marosvári D, Galik B, Rajnai H, Bátai B, et al. Molecular Subtypes and Genomic Profile of Primary Central Nervous System Lymphoma. J Neuropathol Exp Neurol. 2020;79(2):176–83. 10.1093/jnen/nlz125. 31886867

[10] Li S, Young KH, Medeiros LJ. Diffuse large B-cell lymphoma. Pathology. 2018;50(1):74–87. 10.1016/j.pathol.2017.09.006. 29167021

[11] Nakamura T, Tateishi K, Niwa T, Matsushita Y, Tamura K, Kinoshita M, et al. Recurrent mutations of CD79B and MYD88 are the hallmark of primary central nervous system lymphomas. Neuropathol Appl Neurobiol. 2016;42(3):279–90. 10.1111/nan.12259. 26111727

[12] Radke J, Ishaque N, Koll R, Gu Z, Schumann E, Sieverling L, et al. The genomic and transcriptional landscape of primary central nervous system lymphoma. Nat Commun. 2022;13(1):2558. 10.1038/s41467-022-30050-y. PMC9091224 35538064

[13] Klein U, Tu Y, Stolovitzky GA, Keller JL, Haddad J Jr., Miljkovic V, et al. Transcriptional analysis of the B cell germinal center reaction. Proc Natl Acad Sci USA. 2003;100(5):2639–44. 10.1073/pnas.0437996100. PMC151393 12604779

[14] Vater I, Montesinos-Rongen M, Schlesner M, Haake A, Purschke F, Sprute R, et al. The mutational pattern of primary lymphoma of the central nervous system determined by whole-exome sequencing. Leukemia. 2015;29(3):677–85. 10.1038/leu.2014.264. 25189415

[15] Montesinos-Rongen M, Purschke F, Küppers R, Deckert M. Immunoglobulin repertoire of primary lymphomas of the central nervous system. J Neuropathol Exp Neurol. 2014;73(12):1116–25. 10.1097/nen.0000000000000133. 25383641

[16] Cady FM, O’Neill BP, Law ME, Decker PA, Kurtz DM, Giannini C, et al. Del(6)(q22) and BCL6 rearrangements in primary CNS lymphoma are indicators of an aggressive clinical course. J Clin Oncol. 2008;26(29):4814–9. 10.1200/jco.2008.16.1455. PMC2653136 18645192

[17] Courts C, Montesinos-Rongen M, Brunn A, Bug S, Siemer D, Hans V, et al. Recurrent inactivation of the PRDM1 gene in primary central nervous system lymphoma. J Neuropathol Exp Neurol. 2008;67(7):720–7. 10.1097/NEN.0b013e31817dd02d. 18596541

[18] Montesinos-Rongen M, Schäfer E, Siebert R, Deckert M. Genes regulating the B cell receptor pathway are recurrently mutated in primary central nervous system lymphoma. Acta Neuropathol. 2012;124(6):905–6. 10.1007/s00401-012-1064-7. 23138649

[19] Rovida A, Maccalli C, Scarfò L, Dellabona P, Stamatopoulos K, Ghia P. Exploiting B-cell Receptor Stereotypy to Design Tailored Immunotherapy in Chronic Lymphocytic Leukemia. Clin cancer research: official J Am Association Cancer Res. 2021;27(3):729–39. 10.1158/1078-0432.Ccr-20-1632. 33051305

[20] Montesinos-Rongen M, Terrao M, May C, Marcus K, Blümcke I, Hellmich M, et al. The process of somatic hypermutation increases polyreactivity for central nervous system antigens in primary central nervous system lymphoma. Haematologica. 2021;106(3):708–17. 10.3324/haematol.2019.242701. PMC7927892 32193251

[21] Bertoni F, Montesinos-Rongen M. Primary diffuse large B-cell lymphoma of the central nervous system: molecular and biological features of neoplastic cells. Annals Lymphoma. 2022;6. 10.21037/aol-21-38.

[22] Jiang L, Marlow LA, Cooper SJ, Roemeling CV, Menke DM, Copland JA, et al. Selective central nervous system tropism of primary central nervous system lymphoma. Int J Clin Exp Pathol. 2010;3(8):763–7. PMC2993226 21151389

[23] Tun HW, Personett D, Baskerville KA, Menke DM, Jaeckle KA, Kreinest P, et al. Pathway analysis of primary central nervous system lymphoma. Blood. 2008;111(6):3200–10. 10.1182/blood-2007-10-119099. PMC2265457 18184868

[24] Smith JR, Braziel RM, Paoletti S, Lipp M, Uguccioni M, Rosenbaum JT. Expression of B-cell-attracting chemokine 1 (CXCL13) by malignant lymphocytes and vascular endothelium in primary central nervous system lymphoma. Blood. 2003;101(3):815–21. 10.1182/blood-2002-05-1576. 12393412

[25] He M, Zuo C, Wang J, Liu J, Jiao B, Zheng J, et al. Prognostic significance of the aggregative perivascular growth pattern of tumor cells in primary central nervous system diffuse large B-cell lymphoma. Neuro Oncol. 2013;15(6):727–34. 10.1093/neuonc/not012. PMC3661096 23482670

[26] Lemma SA, Kuusisto M, Haapasaari KM, Sormunen R, Lehtinen T, Klaavuniemi T, et al. Integrin alpha 10, CD44, PTEN, cadherin-11 and lactoferrin expressions are potential biomarkers for selecting patients in need of central nervous system prophylaxis in diffuse large B-cell lymphoma. Carcinogenesis. 2017;38(8):812–20. 10.1093/carcin/bgx061. PMC5862348 28854563

[27] Aho R, Kalimo H, Salmi M, Smith D, Jalkanen S. Binding of malignant lymphoid cells to the white matter of the human central nervous system: role of different CD44 isoforms, beta 1, beta 2 and beta 7 integrins, and L-selectin. J Neuropathol Exp Neurol. 1997;56(5):557–68. 10.1097/00005072-199705000-00012. 9143269

[28] Aho R, Jalkanen S, Kalimo H. CD44-hyaluronate interaction mediates in vitro lymphocyte binding to the white matter of the central nervous system. J Neuropathol Exp Neurol. 1994;53(3):295–302. 10.1097/00005072-199405000-00011. 7513749

[29] Venetz D, Ponzoni M, Schiraldi M, Ferreri AJ, Bertoni F, Doglioni C, et al. Perivascular expression of CXCL9 and CXCL12 in primary central nervous system lymphoma: T-cell infiltration and positioning of malignant B cells. Int J Cancer. 2010;127(10):2300–12. 10.1002/ijc.25236. 20872671

[30] Marcelis L, Antoranz A, Delsupehe AM, Biesemans P, Ferreiro JF, Debackere K, et al. In-depth characterization of the tumor microenvironment in central nervous system lymphoma reveals implications for immune-checkpoint therapy. Cancer Immunol immunotherapy: CII. 2020;69(9):1751–66. 10.1007/s00262-020-02575-y. PMC11027603 32335702

[31] Cho H, Kim SH, Kim SJ, Chang JH, Yang WI, Suh CO, et al. The prognostic role of CD68 and FoxP3 expression in patients with primary central nervous system lymphoma. Ann Hematol. 2017;96(7):1163–73. 10.1007/s00277-017-3014-x. 28508176

[32] Jin Q, Jiang H, Han Y, Zhang L, Li C, Zhang Y, et al. Tumor microenvironment in primary central nervous system lymphoma (PCNSL). Cancer Biol Ther. 2024;25(1):2425131. 10.1080/15384047.2024.2425131. PMC11581175 39555697

[33] Rubenstein JL, Fridlyand J, Shen A, Aldape K, Ginzinger D, Batchelor T, et al. Gene expression and angiotropism in primary CNS lymphoma. Blood. 2006;107(9):3716–23. 10.1182/blood-2005-03-0897. PMC1895776 16418334

[34] Montesinos-Rongen M, Purschke FG, Brunn A, May C, Nordhoff E, Marcus K, et al. Primary Central Nervous System (CNS) Lymphoma B Cell Receptors Recognize CNS Proteins. J Immunol (Baltimore Md: 1950). 2015;195(3):1312–9. 10.4049/jimmunol.1402341. 26116512

[35] Nico B, Annese T, Tamma R, Longo V, Ruggieri S, Senetta R et al. Aquaporin-4 expression in primary human central nervous system lymphomas correlates with tumour cell proliferation and phenotypic heterogeneity of the vessel wall. European journal of cancer (Oxford, England: 1990). 2012;48(5):772 – 81. 10.1016/j.ejca.2011.10.022 22100902

[36] Chen R, Zhou D, Wang L, Zhu L, Ye X. MYD88(L265P) and CD79B double mutations type (MCD type) of diffuse large B-cell lymphoma: mechanism, clinical characteristics, and targeted therapy. Ther Adv Hematol. 2022;13:20406207211072839. 10.1177/20406207211072839. PMC8808040 35126963

[37] Mulazzani M, Huber M, Borchard S, Langer S, Angele B, Schuh E, et al. APRIL and BAFF: novel biomarkers for central nervous system lymphoma. J Hematol Oncol. 2019;12(1):102. 10.1186/s13045-019-0796-4. PMC6792247 31615554

[38] Zhou X, Mulazzani M, von Mücke-Heim IA, Langer S, Zhang W, Ishikawa-Ankerhold H, et al. The Role of BAFF-R Signaling in the Growth of Primary Central Nervous System Lymphoma. Front Oncol. 2020;10:682. 10.3389/fonc.2020.00682. PMC7266954 32528875

[39] Mizutani H, Nakane S, Ikeda T, Nakamura H, Takamatsu K, Makino K, et al. CSF TACI and BAFF levels in patients with primary CNS lymphoma as novel diagnostic biomarkers. Ann Clin Transl Neurol. 2018;5(12):1611–6. 10.1002/acn3.668. PMC6292192 30564626

[40] Nam SJ, Kim S, Kwon D, Kim H, Kim S, Lee E, et al. Prognostic implications of tumor-infiltrating macrophages, M2 macrophages, regulatory T-cells, and indoleamine 2,3-dioxygenase-positive cells in primary diffuse large B-cell lymphoma of the central nervous system. Oncoimmunology. 2018;7(7):e1442164. 10.1080/2162402x.2018.1442164. PMC5993494 29900049

[41] Cai F, Wang X, Zhang S, Li Y, Wang S, Zhu J, et al. Single cell profiling reveals malignant states and immune landscapes in PCNSL and systemic DLBCL. iScience. 2026;29(5):115563. 10.1016/j.isci.2026.115563. PMC13097095 42023148

[42] Mosser DM, Zhang X. Interleukin-10: new perspectives on an old cytokine. Immunol Rev. 2008;226:205–18. 10.1111/j.1600-065X.2008.00706.x. PMC2724982 19161426

[43] Mizowaki T, Sasayama T, Tanaka K, Mizukawa K, Takata K, Nakamizo S, et al. STAT3 activation is associated with cerebrospinal fluid interleukin-10 (IL-10) in primary central nervous system diffuse large B cell lymphoma. J Neurooncol. 2015;124(2):165–74. 10.1007/s11060-015-1843-9. 26080800

[44] Sasayama T, Nakamizo S, Nishihara M, Kawamura A, Tanaka H, Mizukawa K, et al. Cerebrospinal fluid interleukin-10 is a potentially useful biomarker in immunocompetent primary central nervous system lymphoma (PCNSL). Neurooncology. 2012;14(3):368–80. 10.1093/neuonc/nor203. PMC3280797 22156547

[45] Furuse M, Kuwabara H, Ikeda N, Hattori Y, Ichikawa T, Kagawa N, et al. PD-L1 and PD-L2 expression in the tumor microenvironment including peritumoral tissue in primary central nervous system lymphoma. BMC Cancer. 2020;20(1):277. 10.1186/s12885-020-06755-y. PMC7132991 32248797

[46] Miyasato Y, Takashima Y, Takeya H, Yano H, Hayano A, Nakagawa T, et al. The expression of PD-1 ligands and IDO1 by macrophage/microglia in primary central nervous system lymphoma. J Clin Exp Hematop. 2018;58(2):95–101. 10.3960/jslrt.18001. PMC6413151 29998979

[47] Abdulla M, Alexsson A, Sundström C, Ladenvall C, Mansouri L, Lindskog C, et al. PD-L1 and IDO1 are potential targets for treatment in patients with primary diffuse large B-cell lymphoma of the CNS. Acta Oncol. 2021;60(4):531–8. 10.1080/0284186x.2021.1881161. 33579170

[48] Hong L, Liu M, Sridhar S, Ong C, Jennifer B, Hamberger F, et al. Spatial multi-omics reveal distinct immunosuppressive lipid-laden macrophages in primary CNS lymphoma compared to systemic DLBCL. Blood. 2025;146(Supplement 1):146. 10.1182/blood-2025-146.

[49] Riemersma SA, Oudejans JJ, Vonk MJ, Dreef EJ, Prins FA, Jansen PM, et al. High numbers of tumour-infiltrating activated cytotoxic T lymphocytes, and frequent loss of HLA class I and II expression, are features of aggressive B cell lymphomas of the brain and testis. J Pathol. 2005;206(3):328–36. 10.1002/path.1783. 15887291

[50] Farhood B, Najafi M, Mortezaee K. CD8(+) cytotoxic T lymphocytes in cancer immunotherapy: A review. J Cell Physiol. 2019;234(6):8509–21. 10.1002/jcp.27782. 30520029

[51] Fangazio M, Ladewig E, Gomez K, Garcia-Ibanez L, Kumar R, Teruya-Feldstein J, et al. Genetic mechanisms of HLA-I loss and immune escape in diffuse large B cell lymphoma. Proc Natl Acad Sci USA. 2021;118(22). 10.1073/pnas.2104504118. PMC8179151 34050029

[52] Gravelle P, Burroni B, Péricart S, Rossi C, Bezombes C, Tosolini M, et al. Mechanisms of PD-1/PD-L1 expression and prognostic relevance in non-Hodgkin lymphoma: a summary of immunohistochemical studies. Oncotarget. 2017;8(27):44960–75. 10.18632/oncotarget.16680. PMC5546533 28402953

[53] Sugita Y, Furuta T, Ohshima K, Komaki S, Miyoshi J, Morioka M, et al. The perivascular microenvironment in Epstein-Barr virus positive primary central nervous system lymphoma: The role of programmed cell death 1 and programmed cell death ligand 1. Neuropathology: official J Japanese Soc Neuropathology. 2018;38(2):125–34. 10.1111/neup.12435. 29067721

[54] Nishii S, Akatani R, Shiroma K, Takeda R, Tsuji A, Katanazaka K, et al. Soluble TIM-3 and galectin-9 serve as additional diagnostic biomarkers in primary central nervous system lymphoma. J Neurol. 2026;273(4). 10.1007/s00415-026-13791-4. PMC13046686 41927833

[55] Dutoit V, Migliorini D, Dietrich PY, Walker PR. Immunotherapy of Malignant Tumors in the Brain: How Different from Other Sites? Front Oncol. 2016;6:256. 10.3389/fonc.2016.00256. PMC5141244 28003994

[56] Bechmann I, Mor G, Nilsen J, Eliza M, Nitsch R, Naftolin F. FasL (CD95L, Apo1L) is expressed in the normal rat and human brain: evidence for the existence of an immunological brain barrier. Glia. 1999;27(1):62–74. 10.1002/(sici)1098-1136(199907)27:1<62::aid-glia7>3.0.co;2-s. 10401633

[57] Petty AJ, Yang Y. Tumor-associated macrophages in hematologic malignancies: new insights and targeted therapies. Cells. 2019;8(12). 10.3390/cells8121526. PMC6952752 31783588

[58] Yamaguchi J, Ohka F, Lushun C, Motomura K, Aoki K, Takeuchi K, et al. CD79B Y196 mutation is a potent predictive marker for favorable response to R-MPV in primary central nervous system lymphoma. Cancer Med. 2023;12(6):7116–26. 10.1002/cam4.5512. PMC10067082 36478416

[59] Nayyar N, White MD, Gill CM, Lastrapes M, Bertalan M, Kaplan A, et al. MYD88 L265P mutation and CDKN2A loss are early mutational events in primary central nervous system diffuse large B-cell lymphomas. Blood Adv. 2019;3(3):375–83. 10.1182/bloodadvances.2018027672. PMC6373750 30723112

[60] Ferreri AJM, Calimeri T, Lopedote P, Francaviglia I, Daverio R, Iacona C, et al. MYD88 L265P mutation and interleukin-10 detection in cerebrospinal fluid are highly specific discriminating markers in patients with primary central nervous system lymphoma: results from a prospective study. Br J Haematol. 2021;193(3):497–505. 10.1111/bjh.17357. 33620087

[61] Miserocchi E, Ferreri AJM, Giuffrè C, Cangi MG, Francaviglia I, Calimeri T, MYD88 L265P MUTATION DETECTION IN THE AQUEOUS HUMOR OF PATIENTS WITH VITREORETINAL LYMPHOMA, et al. Retina. 2019;39(4):679–84. 10.1097/iae.0000000000002319. 30204732

[62] Barakat M, Albitar M, Whitney R, Abdulhaq H. Diversity of genetic alterations of primary central nervous system lymphoma in Hispanic versus non-Hispanic patients. Cancer Treat Res Commun. 2021;27:100310. 10.1016/j.ctarc.2021.100310. 33581493

[63] Montesinos-Rongen M, Schmitz R, Brunn A, Gesk S, Richter J, Hong K, et al. Mutations of CARD11 but not TNFAIP3 may activate the NF-kappaB pathway in primary CNS lymphoma. Acta Neuropathol. 2010;120(4):529–35. 10.1007/s00401-010-0709-7. 20544211

[64] Gonzalez-Aguilar A, Idbaih A, Boisselier B, Habbita N, Rossetto M, Laurenge A, et al. Recurrent mutations of MYD88 and TBL1XR1 in primary central nervous system lymphomas. Clin Cancer Res. 2012;18(19):5203–11. 10.1158/1078-0432.Ccr-12-0845. 22837180

[65] Chapuy B, Kamburov A, Coughlin CA, Stewart C, Dunford A, Aono M, et al. In Silico and Functional Characterization of TBL1XR1 as a Tumor Suppressor in Large B-Cell Lymphomas. Blood. 2016;128(22):612. 10.1182/blood.V128.22.612.612.

[66] Gindin T, Hussein S. Special sites: primary mediastinal large B-cell lymphoma, primary CNS diffuse large B-cell lymphoma, and primary testicular diffuse large B-cell lymphoma. precision molecular pathology of aggressive B-cell lymphomas. Springer; 2024. pp. 221–43.

[67] Pasqualucci L, Klein U. NF-κB mutations in germinal center B-cell lymphomas: relation to NF-κB function in normal B cells. Biomedicines. 2022;10(10). 10.3390/biomedicines10102450. PMC9599362 36289712

[68] Kaulen LD, Erson-Omay EZ, Henegariu O, Karschnia P, Huttner A, Günel M, et al. P11.46.A Whole exome sequencing identifies novel SLIT2 mutations in primary CNS lymphoma. Neuro Oncol. 2022;24(Suppl 2):ii68. 10.1093/neuonc/noac174.235. eCollection 2022 Sep.

[69] Li S, Li D, Xia Z, Wu J, Ren J, Li Y, et al. Genomic landscape and molecular subtypes of primary central nervous system lymphoma. Blood Cancer J. 2025;16(1):8. 10.1038/s41408-025-01421-7. PMC12783669 41298346

[70] Takashima Y, Sasaki Y, Hayano A, Homma J, Fukai J, Iwadate Y, et al. Target amplicon exome-sequencing identifies promising diagnosis and prognostic markers involved in RTK-RAS and PI3K-AKT signaling as central oncopathways in primary central nervous system lymphoma. Oncotarget. 2018;9(44):27471–86. 10.18632/oncotarget.25463. PMC6007945 29937999

[71] Schwindt H, Vater I, Kreuz M, Montesinos-Rongen M, Brunn A, Richter J, et al. Chromosomal imbalances and partial uniparental disomies in primary central nervous system lymphoma. Leukemia. 2009;23(10):1875–84. 10.1038/leu.2009.120. 19494841

[72] Zorofchian S, El-Achi H, Yan Y, Esquenazi Y, Ballester LY. Characterization of genomic alterations in primary central nervous system lymphomas. J Neurooncol. 2018;140(3):509–17. 10.1007/s11060-018-2990-6. 30171453

[73] Vallée A, Lecarpentier Y, Vallée JN. Hypothesis of Opposite Interplay Between the Canonical WNT/beta-catenin Pathway and PPAR Gamma in Primary Central Nervous System Lymphomas. Curr Issues Mol Biol. 2019;31:1–20. 10.21775/cimb.031.001. 30647194

[74] Montesinos-Rongen M, Van Roost D, Schaller C, Wiestler OD, Deckert M. Primary diffuse large B-cell lymphomas of the central nervous system are targeted by aberrant somatic hypermutation. Blood. 2004;103(5):1869–75. 10.1182/blood-2003-05-1465. 14592832

[75] Bruno A, Boisselier B, Labreche K, Marie Y, Polivka M, Jouvet A, et al. Mutational analysis of primary central nervous system lymphoma. Oncotarget. 2014;5(13):5065–75. 10.18632/oncotarget.2080. PMC4148122 24970810

[76] Nakamura M, Sakaki T, Hashimoto H, Nakase H, Ishida E, Shimada K, et al. Frequent alterations of the p14(ARF) and p16(INK4a) genes in primary central nervous system lymphomas. Cancer Res. 2001;61(17):6335–9. 11522621

[77] Baraniskin A, Kuhnhenn J, Schlegel U, Chan A, Deckert M, Gold R, et al. Identification of microRNAs in the cerebrospinal fluid as marker for primary diffuse large B-cell lymphoma of the central nervous system. Blood. 2011;117(11):3140–6. 10.1182/blood-2010-09-308684. 21200023

[78] Robertus JL, Harms G, Blokzijl T, Booman M, de Jong D, van Imhoff G, et al. Specific expression of miR-17-5p and miR-127 in testicular and central nervous system diffuse large B-cell lymphoma. Mod pathology: official J United States Can Acad Pathol Inc. 2009;22(4):547–55. 10.1038/modpathol.2009.10. 19287466

[79] Baraniskin A, Kuhnhenn J, Schlegel U, Schmiegel W, Hahn S, Schroers R. MicroRNAs in cerebrospinal fluid as biomarker for disease course monitoring in primary central nervous system lymphoma. J Neurooncol. 2012;109(2):239–44. 10.1007/s11060-012-0908-2. 22729947

[80] Baraniskin A, Chomiak M, Ahle G, Gress T, Buchholz M, Turewicz M, et al. MicroRNA-30c as a novel diagnostic biomarker for primary and secondary B-cell lymphoma of the CNS. J Neurooncol. 2018;137(3):463–8. 10.1007/s11060-018-2749-0. 29327175

[81] Takashima Y, Yoshimura T, Kano Y, Hayano A, Hondoh H, Ikenaka K, et al. Differential expression of N-linked oligosaccharides in methotrexate-resistant primary central nervous system lymphoma cells. BMC Cancer. 2019;19(1):910. 10.1186/s12885-019-6129-8. PMC6739943 31510952

[82] Hai L, Friedel D, Hinz F, Hoffmann DC, Doubrovinskaia S, Rohdjess H, et al. Distinct epigenetic and transcriptional profiles of Epstein-Barr virus-positive and negative primary CNS lymphomas. Neurooncology. 2025;27(4):979–92. 10.1093/neuonc/noae251. PMC12083237 39575767

[83] Chapuy B, Roemer MG, Stewart C, Tan Y, Abo RP, Zhang L, et al. Targetable genetic features of primary testicular and primary central nervous system lymphomas. Blood. 2016;127(7):869–81. 10.1182/blood-2015-10-673236. PMC4760091 26702065

[84] Mao H, Zhao X, Sun SC. NF-κB in inflammation and cancer. Cell Mol Immunol. 2025;22(8):811–39. 10.1038/s41423-025-01310-w. PMC12310982 40562870

[85] Cao J, Xiong S, Zhang S, Yue N, Wu C, Targeting. PD-L1 for PCNS-DLBCL: from molecular effects to clinical translation. Front Immunol. 2025;16:1647045. 10.3389/fimmu.2025.1647045. PMC12510830 41080552

[86] Berghoff AS, Ricken G, Widhalm G, Rajky O, Hainfellner JA, Birner P, et al. PD1 (CD279) and PD-L1 (CD274, B7H1) expression in primary central nervous system lymphomas (PCNSL). Clin Neuropathol. 2014;33(1):42–9. 10.5414/np300698. 24359606

[87] Four M, Cacheux V, Tempier A, Platero D, Fabbro M, Marin G, et al. PD1 and PDL1 expression in primary central nervous system diffuse large B-cell lymphoma are frequent and expression of PD1 predicts poor survival. Hematol Oncol. 2017;35(4):487–96. 10.1002/hon.2375. 27966264

[88] Gandhi MK, Hoang T, Law SC, Brosda S, O’Rourke K, Tobin JWD, et al. EBV-associated primary CNS lymphoma occurring after immunosuppression is a distinct immunobiological entity. Blood. 2021;137(11):1468–77. 10.1182/blood.2020008520. PMC7976507 33202420

[89] Kreisl TN, Panageas KS, Elkin EB, Deangelis LM, Abrey LE. Treatment patterns and prognosis in patients with human immunodeficiency virus and primary central system lymphoma. Leuk Lymphoma. 2008;49(9):1710–6. 10.1080/10428190802238560. 18661394

[90] Kaulen LD, Denisova E, Hinz F, Hai L, Friedel D, Henegariu O, et al. Integrated genetic analyses of immunodeficiency-associated Epstein-Barr virus- (EBV) positive primary CNS lymphomas. Acta Neuropathol. 2023;146(3):499–514. 10.1007/s00401-023-02613-w. PMC10412493 37495858

[91] Gasser O, Bihl FK, Wolbers M, Loggi E, Steffen I, Hirsch HH, et al. HIV patients developing primary CNS lymphoma lack EBV-specific CD4 + T cell function irrespective of absolute CD4 + T cell counts. PLoS Med. 2007;4(3):e96. 10.1371/journal.pmed.0040096. PMC1831733 17388662

[92] Li M, Ding X, Liang B, Chen J, Zhou X, Feng E. The activation of the NF-κB p65/PD-L1 signaling pathway in AIDS-related PCNSL is related to TNF-α and IFN-γ but not to MYD88 and CD79B mutations. Pathol Res Pract. 2025;272:156050. 10.1016/j.prp.2025.156050. 40450975

[93] Kim B, Chae SW, Kim HJ, Roh J, Lee YJ, Jo JC, et al. Comparative analysis of the tumor microenvironment in primary CNS and testicular large B-cell lymphomas using digital image analysis and its implications for immunotherapy. Hum Pathol. 2025;163:105874. 10.1016/j.humpath.2025.105874. 40714128

[94] Pagès-Geli C, Medina-Gil D, Fernández-Guzmán P, Hernández C, Caràbia J, Pujadas G, et al. Immune evasion in primary central nervous system lymphoma: macrophage and T cell roles in response to immunotherapy. Haematologica. 2026. 10.3324/haematol.2025.300015.42021682

[95] Liu Y, Tan H, Dai J, Lin J, Zhao K, Hu H, et al. Targeting macrophages in cancer immunotherapy: Frontiers and challenges. J Adv Res. 2025;76:695–713. 10.1016/j.jare.2024.12.043. PMC12793739 39778768

[96] Cornice J, Verzella D, Arboretto P, Vecchiotti D, Capece D, Zazzeroni F, et al. NF-κB: governing macrophages in cancer. Genes (Basel). 2024;15(2). 10.3390/genes15020197. PMC10888451 38397187

[97] Galtier J, Peanne R, Le Morvan V, Blondy T, Melane M, Bidon A, et al. Cutaneous diffuse large B-cell lymphoma induces a macrophage immunosuppressive phenotype through IL-10 secretion. Blood Adv. 2026;10(3):850–63. 10.1182/bloodadvances.2025016492. PMC12887783 41358745

[98] Xu J, Ding L, Mei J, Hu Y, Kong X, Dai S, et al. Dual roles and therapeutic targeting of tumor-associated macrophages in tumor microenvironments. Signal Transduct Target Ther. 2025;10(1):268. 10.1038/s41392-025-02325-5. PMC12375796 40850976

[99] Komohara Y, Horlad H, Ohnishi K, Ohta K, Makino K, Hondo H, et al. M2 macrophage/microglial cells induce activation of Stat3 in primary central nervous system lymphoma. J Clin Exp Hematop. 2011;51(2):93–9. 10.3960/jslrt.51.93. 22104307

[100] Qiu H, Gong S, Xu L, Cheng H, Gao L, Chen J, et al. MYD88 L265P mutation promoted malignant B cell resistance against T cell-mediated cytotoxicity via upregulating the IL-10/STAT3 cascade. Int Immunopharmacol. 2018;64:394–400. 10.1016/j.intimp.2018.09.024. 30253331

[101] Ho P, Melms JC, Rogava M, Frangieh CJ, Poźniak J, Shah SB, et al. The CD58-CD2 axis is co-regulated with PD-L1 via CMTM6 and shapes anti-tumor immunity. Cancer Cell. 2023;41(7):1207–e2112. 10.1016/j.ccell.2023.05.014. PMC10524902 37327789

[102] Alame M, Cornillot E, Cacheux V, Rigau V, Costes-Martineau V, Lacheretz-Szablewski V, et al. The immune contexture of primary central nervous system diffuse large B cell lymphoma associates with patient survival and specific cell signaling. Theranostics. 2021;11(8):3565–79. 10.7150/thno.54343. PMC7914352 33664848

[103] Wang HC, Hsiao HH, Du JS, Cho SF, Yeh TJ, Gau YC, et al. Effect of Tumor Microenvironment and Angiogenesis on Clinical Outcomes of Primary Central Nervous System Lymphoma. Biomed Res Int. 2021;2021:3291762. 10.1155/2021/3291762. PMC8497102 34631879

[104] Kitamura T, Qian BZ, Pollard JW. Immune cell promotion of metastasis. Nat Rev Immunol. 2015;15(2):73–86. 10.1038/nri3789. PMC4470277 25614318

[105] Quail DF, Joyce JA. The Microenvironmental Landscape of Brain Tumors. Cancer Cell. 2017;31(3):326–41. 10.1016/j.ccell.2017.02.009. PMC5424263 28292436

[106] Jiang H, Nong L. The Landscape of Primary Central Nervous System Lymphoma (PCNSL): clinicopathologic and genomic characteristics and therapeutic perspectives. Cancers (Basel). 2025;17(17). 10.3390/cancers17172909. PMC12428670 40941006

[107] Fan Y, Mao R, Yang J. NF-κB and STAT3 signaling pathways collaboratively link inflammation to cancer. Protein Cell. 2013;4(3):176–85. 10.1007/s13238-013-2084-3. PMC4875500 23483479

[108] He M, Zhang W, Wang J, Gao L, Jiao L, Wang L, et al. Aggregative Perivascular Tumor Cell Growth Pattern of Primary Central Nervous System Lymphomas Is Associated with Hypoxia-Related Endoplasmic Reticulum Stress. J Cancer. 2021;12(13):3841–52. 10.7150/jca.54952. PMC8176238 34093792

[109] Ma J, Lin Z, Zhang Y, Ding Y, Tang Q, Qian Y, et al. Targeted multiplex validation of CSF proteomic biomarkers: implications for differentiation of PCNSL from tumor-free controls and other brain tumors. Front Immunol. 2024;15:1343109. 10.3389/fimmu.2024.1343109. PMC11322575 39144147

[110] Domènech M, Hernández A, Plaja A, Martínez-Balibrea E, Balañà C. Hypoxia: the cornerstone of glioblastoma. Int J Mol Sci. 2021;22(22). 10.3390/ijms222212608. PMC8620858 34830491

[111] Shen N, Wang Y, Sun X, Bai X, He J, Cui Q, et al. Expression of hypoxia-inducible factor 1α, glucose transporter 1, and hexokinase 2 in primary central nervous system lymphoma and the correlation with the biological behaviors. Brain Behav. 2020;10(8):e01718. 10.1002/brb3.1718. PMC7428508 32533646

[112] Semenza GL. HIF-1 mediates metabolic responses to intratumoral hypoxia and oncogenic mutations. J Clin Invest. 2013;123(9):3664–71. 10.1172/jci67230. PMC3754249 23999440

[113] Semenza GL, Jiang BH, Leung SW, Passantino R, Concordet JP, Maire P, et al. Hypoxia response elements in the aldolase A, enolase 1, and lactate dehydrogenase A gene promoters contain essential binding sites for hypoxia-inducible factor 1. J Biol Chem. 1996;271(51):32529–37. 10.1074/jbc.271.51.32529. 8955077

[114] Yang Y, Wu Y, Chen H, Xu Z, Lu R, Zhang S, et al. Research progress on the interaction between glucose metabolic reprogramming and lactylation in tumors. Front Immunol. 2025;16:1595162. 10.3389/fimmu.2025.1595162. PMC12315337 40755753

[115] Ajam-Hosseini M, Heydari R, Rasouli M, Akhoondi F, Asadi Hanjani N, Bekeschus S, et al. Lactic acid in macrophage polarization: A factor in carcinogenesis and a promising target for cancer therapy. Biochem Pharmacol. 2024;222:116098. 10.1016/j.bcp.2024.116098. 38431231

[116] Jin X, Zhang N, Yan T, Wei J, Hao L, Sun C, et al. Lactate-mediated metabolic reprogramming of tumor-associated macrophages: implications for tumor progression and therapeutic potential. Front Immunol. 2025;16:1573039. 10.3389/fimmu.2025.1573039. PMC12106438 40433363

[117] Valle-Mendiola A, Soto-Cruz I. Energy metabolism in cancer: the roles of STAT3 and STAT5 in the regulation of metabolism-related genes. Cancers (Basel). 2020;12(1). 10.3390/cancers12010124. PMC7016889 31947710

[118] Zhang X, Wu Y, Sun X, Cui Q, Bai X, Dong G, et al. The PI3K/AKT/mTOR signaling pathway is aberrantly activated in primary central nervous system lymphoma and correlated with a poor prognosis. BMC Cancer. 2022;22(1):190. 10.1186/s12885-022-09275-z. PMC8859899 35184749

[119] Zhou L, Li Q, Xu J, Wang S, Song Z, Chen X, et al. Cerebrospinal fluid metabolic markers predict prognosis behavior of primary central nervous system lymphoma with high-dose methotrexate-based chemotherapeutic treatment. Neurooncol Adv. 2023;5(1):vdac181. 10.1093/noajnl/vdac181. PMC9985165 36879663

[120] Zhong Y, Zhou L, Xu J, Huang H. Predicting prognosis outcomes of primary central nervous system lymphoma with high-dose methotrexate-based chemotherapeutic treatment using lipidomics. Neurooncol Adv. 2024;6(1):vdae119. 10.1093/noajnl/vdae119. PMC11306931 39119277

[121] Los-de Vries GT, Stathi P, Rutkens R, Hijmering NJ, Luijks J, Groenen P, et al. Large B-cell Lymphomas of Immune-Privileged Sites Relapse via Parallel Clonal Evolution from a Common Progenitor B Cell. Cancer Res. 2023;83(11):1917–27. 10.1158/0008-5472.Can-22-3814. 36971477

[122] Heming M, Haessner S, Wolbert J, Lu IN, Li X, Brokinkel B, et al. Intratumor heterogeneity and T cell exhaustion in primary CNS lymphoma. Genome Med. 2022;14(1):109. 10.1186/s13073-022-01110-1. PMC9509601 36153593

[123] Xia Y, Sun T, Li G, Li M, Wang D, Su X, et al. Spatial single cell analysis of tumor microenvironment remodeling pattern in primary central nervous system lymphoma. Leukemia. 2023;37(7):1499–510. 10.1038/s41375-023-01908-x. PMC10317840 37120690

[124] Zhong L, Lu A, Lu X, Liu X, Cao L, Zhu S, et al. Molecular Mechanisms and Therapeutic Prospects of Immunotherapy and Targeted Therapy in Primary Central Nervous System Lymphoma. Technol Cancer Res Treat. 2025;24:15330338251319394. 10.1177/15330338251319394. PMC11800258 39912261

[125] Ariav Y, Ch’ng JH, Christofk HR, Ron-Harel N, Erez A. Targeting nucleotide metabolism as the nexus of viral infections, cancer, and the immune response. Sci Adv. 2021;7(21). 10.1126/sciadv.abg6165. PMC8133749 34138729

[126] Madsen HB, Peeters MJ, Straten PT, Desler C. Nucleotide metabolism in the regulation of tumor microenvironment and immune cell function. Curr Opin Biotechnol. 2023;84:103008. 10.1016/j.copbio.2023.103008. 37863018

[127] Xu X, Zhang Y, Lu Y, Zhang X, Zhao C, Wang J, et al. CD58 Alterations Govern Antitumor Immune Responses by Inducing PDL1 and IDO in Diffuse Large B-Cell Lymphoma. Cancer Res. 2024;84(13):2123–40. 10.1158/0008-5472.Can-23-2874. 38635903

[128] Yan X, Chen D, Ma X, Wang Y, Guo Y, Wei J, et al. CD58 loss in tumor cells confers functional impairment of CAR T cells. Blood Adv. 2022;6(22):5844–56. 10.1182/bloodadvances.2022007891. PMC9649996 35728062

[129] Carturan A, Angelos MG, Guruprasad P, Patel RP, Pajarillo R, Lee A, et al. Harnessing the CD2 axis to broaden and enhance the efficacy of CAR T-cell therapies. Blood. 2026;147(16):1842–56. 10.1182/blood.2025031409. PMC13181175 41490267

[130] Romain G, Strati P, Rezvan A, Fathi M, Bandey IN, Adolacion JRT, et al. Multidimensional single-cell analysis identifies a role for CD2-CD58 interactions in clinical antitumor T cell responses. J Clin Invest. 2022;132(17). 10.1172/jci159402. PMC9433104 35881486

[131] Nayak L, Iwamoto FM, LaCasce A, Mukundan S, Roemer MGM, Chapuy B, et al. PD-1 blockade with nivolumab in relapsed/refractory primary central nervous system and testicular lymphoma. Blood. 2017;129(23):3071–3. 10.1182/blood-2017-01-764209. PMC5766844 28356247

[132] Hoang-Xuan K, Houot R, Soussain C, Blonski M, Schmitt A, Delwail V, et al. First Results of the Acsé Pembrolizumab Phase II in the Primary CNS Lymphoma (PCNSL) Cohort. Blood. 2020;136(Supplement 1):15–6. 10.1182/blood-2020-141773.

[133] Lacan C, Caron J, Tarantino N, Fouquet B, Cherai M, Parizot C, et al. CAR T-cell therapy for central nervous system lymphomas: blood and cerebrospinal fluid biology, and outcomes. Haematologica. 2023;108(12):3485–90. 10.3324/haematol.2023.282875. PMC10690903 37345469

[134] Hu Y, Sun J, Wu Z, Yu J, Cui Q, Pu C, et al. Predominant cerebral cytokine release syndrome in CD19-directed chimeric antigen receptor-modified T cell therapy. J Hematol Oncol. 2016;9(1):70. 10.1186/s13045-016-0299-5. PMC4986179 27526682

[135] Li X, Da H, Zhao J, Yan Y, Wei J, Tian W. Breakthroughs and challenges of CAR-T cell therapy in treating hematologic malignancies of the central nervous system. Blood Sci. 2026;8(2):e00289. 10.1097/bs9.0000000000000289. PMC13124383 42064783

[136] Frigault MJ, Dietrich J, Gallagher K, Roschewski M, Jordan JT, Forst D, et al. Safety and efficacy of tisagenlecleucel in primary CNS lymphoma: a phase 1/2 clinical trial. Blood. 2022;139(15):2306–15. 10.1182/blood.2021014738. PMC9012129 35167655

[137] Cook MR, Dorris CS, Makambi KH, Luo Y, Munshi PN, Donato M, et al. Toxicity and efficacy of CAR T-cell therapy in primary and secondary CNS lymphoma: a meta-analysis of 128 patients. Blood Adv. 2023;7(1):32–9. 10.1182/bloodadvances.2022008525. PMC9813524 36260735

[138] Alcantara M, Houillier C, Blonski M, Rubio MT, Willems L, Rascalou AW, et al. CAR T-cell therapy in primary central nervous system lymphoma: the clinical experience of the French LOC network. Blood. 2022;139(5):792–6. 10.1182/blood.2021012932. PMC8814680 34871363

[139] Choquet S, Soussain C, Azar N, Morel V, Metz C, Ursu R, et al. CAR T-cell therapy induces a high rate of prolonged remission in relapsed primary CNS lymphoma: Real-life results of the LOC network. Am J Hematol. 2024;99(7):1240–9. 10.1002/ajh.27316. 38586986

[140] Ossami Saidy A, Peczynski C, Thieblemont C, Daskalakis M, Wehrli M, Beauvais D, et al. Efficacy and safety of CAR T-cell therapy in patients with primary or secondary CNS lymphoma: A study on behalf of the EBMT and the GoCART coalition. Hemasphere. 2025;9(5):e70146. 10.1002/hem3.70146. PMC12093105 40400509

[141] Zhou X, Yu Q, Dai Z, Wang J, Li C, Huang L, et al. CD19/CD22 CAR-T-cell cocktail therapy following autologous transplantation is an optimizing strategy for treating relapsed/refractory central nervous system lymphoma. Exp Hematol Oncol. 2024;13(1):100. 10.1186/s40164-024-00538-y. PMC11471030 39397022

[142] Honigberg LA, Smith AM, Sirisawad M, Verner E, Loury D, Chang B, et al. The Bruton tyrosine kinase inhibitor PCI-32765 blocks B-cell activation and is efficacious in models of autoimmune disease and B-cell malignancy. Proc Natl Acad Sci U S A. 2010;107(29):13075–80. 10.1073/pnas.1004594107. PMC2919935 20615965

[143] Herman SE, Mustafa RZ, Gyamfi JA, Pittaluga S, Chang S, Chang B, et al. Ibrutinib inhibits BCR and NF-κB signaling and reduces tumor proliferation in tissue-resident cells of patients with CLL. Blood. 2014;123(21):3286–95. 10.1182/blood-2014-02-548610. PMC4046423 24659631

[144] Dubovsky JA, Beckwith KA, Natarajan G, Woyach JA, Jaglowski S, Zhong Y, et al. Ibrutinib is an irreversible molecular inhibitor of ITK driving a Th1-selective pressure in T lymphocytes. Blood. 2013;122(15):2539–49. 10.1182/blood-2013-06-507947. PMC3795457 23886836

[145] Li S, Lu J, Kang Z, Yang H, Qian W, Chen X, et al. Design, preclinical evaluation, and multicenter phase 1 clinical study of HZ-A-018 for relapsed or refractory central nervous system lymphoma. Acta Pharm Sin B. 2026;16(8):5351–62. 10.1016/j.apsb.2026.05.021. PMC13464085 42592300

[146] Jiménez I, Carabia J, Bobillo S, Palacio C, Abrisqueta P, Pagès C, et al. Repolarization of tumor infiltrating macrophages and increased survival in mouse primary CNS lymphomas after XPO1 and BTK inhibition. J Neurooncol. 2020;149(1):13–25. 10.1007/s11060-020-03580-y. PMC7452938 32691208

[147] Xia W, Zhang X, Wang Y, Huang Z, Guo X, Fang L. Progress in targeting tumor-associated macrophages in cancer immunotherapy. Front Immunol. 2025;16:1658795. 10.3389/fimmu.2025.1658795. PMC12420277 40936918

[148] Rubenstein JL, Geng H, Fraser EJ, Formaker P, Chen L, Sharma J, et al. Phase 1 investigation of lenalidomide/rituximab plus outcomes of lenalidomide maintenance in relapsed CNS lymphoma. Blood Adv. 2018;2(13):1595–607. 10.1182/bloodadvances.2017014845. PMC6039666 29986852

[149] Schaff LR, Piotrowski AF, Pentsova E, Gavrilovic IT, Lin A, Kaley TJ, et al. Phase Ib study with expansion of ibrutinib, lenalidomide, and rituximab in patients with relapsed/refractory central nervous system lymphoma. Neuro Oncol. 2025;27(8):2107–16. 10.1093/neuonc/noaf104. PMC12448882 40269592

[150] Yonezawa H, Narita Y, Nagane M, Mishima K, Terui Y, Arakawa Y, et al. Three-year follow-up analysis of phase 1/2 study on tirabrutinib in patients with relapsed or refractory primary central nervous system lymphoma. Neurooncol Adv. 2024;6(1):vdae037. 10.1093/noajnl/vdae037. PMC11059299 38690230

[151] Tun HW, Johnston PB, DeAngelis LM, Atherton PJ, Pederson LD, Koenig PA, et al. Phase 1 study of pomalidomide and dexamethasone for relapsed/refractory primary CNS or vitreoretinal lymphoma. Blood. 2018;132(21):2240–8. 10.1182/blood-2018-02-835496. PMC6265643 30262659

